# Using active learning methodologies to teach sequence analysis and molecular phylogeny

**DOI:** 10.1002/bmb.21861

**Published:** 2024-10-14

**Authors:** Inmaculada Ortiz Martín, Ángel Del Espino Pérez, Estefanía García Luque, Enrique Viguera Mínguez

**Affiliations:** ^1^ Department of Cellular Biology, Genetics and Physiology, Faculty of Science University of Malaga Malaga Spain

**Keywords:** active learning methods, bioinformatics, molecular databases, molecular phylogeny

## Abstract

The great development of high‐throughput molecular biology techniques and the consequent generation of massive data have made Bioinformatics essential for undergraduate Bioscience students. The importance of this scientific discipline is evidenced by the huge number of specialized publications, tools, and databases available. Training in Bioinformatics equips undergraduates with transferable skills that can be applied in all fields of Biology, such as programming abilities, data analysis, database management, biological knowledge, statistics, problem solving, and interdisciplinary collaboration. Over the past decade, there has been a notable increase in the number of higher education institutions worldwide that have adopted a competency‐based curricula. This approach places a significant emphasis on the actions and skills that students are expected to develop, rather than merely focusing on the information, they are required to memorize. In this educational context, the use of active learning strategies has been demonstrated to enhance student comprehension and competency development. This paper describes the implementation of an active learning approach in a hands‐on lesson performed by undergraduate students of Biology at the University of Malaga (Spain). Its main objective is to introduce students to molecular databases and information search systems on genes, proteins, and phylogeny. This is achieved within the framework of a smart campus, which integrates technological and sustainable resources to promote a positive and productive learning environment for the university community. This work presents the content and procedure of this practical activity, as well as the evaluation method and the results of a student survey to assess their opinions.

## 1 INTRODUCTION

The main interest in the study of biology is to understand life itself, which implies finding out about its origin and evolution. In this context, molecular biology and genetics play a central role in structuring and unifying the study of these biological phenomena, since the genetic variability of a population determines its ability to adapt to environmental changes and, consequently, its evolutionary success. Current biological diversity is therefore the result of a process of change from the ancestors that occurs at different levels. While at the population level, the result of this evolutionary process is speciation, at the molecular level it is the modification of the gene pool of a population. This genetic alteration is the result of sequence variation mechanisms, such as mutation and duplication.^1^

Molecular phylogeny studies the evolutionary relationships among organisms by analyzing their sequence similarities and/or differences. It is a recent discipline that has emerged from the development of high‐throughput omics technologies, which have provided a huge amount of sequence information. The phylogenetic analysis of homologous DNA and protein molecules in different species, named orthologs, requires the use of biocomputational applications for sequence alignment and phylogenetic tree construction to assess the evolutionary divergence of sequences and, by extension, of species. It is broadly accepted that the number of sequence substitutions is proportional to the evolutionary time. In other words, the rate of molecular evolution is nearly constant over time.^2^ Although both nucleotide acids and proteins are used as evolutionary clocks (which is the name given to the molecules that help determine when species diverged from a common ancestor), phylogenetic analysis of DNA sequences may be more informative due to the genetic code is degenerate and, thus, some nucleotide variations are silent without amino acid replacements.

Lectures in informatics enable the first‐year undergraduate biology students to learn the management of the basic bioinformatics tools used in the field of life sciences, such as on‐line molecular databases and computer programs. Therefore, this subject offers an eminently practical approach with the aim of providing students with the knowledge and skills whose application and development will be useful in their academic future. At the University of Malaga (UMA), the subject of Informatics comprises three blocks with contents of computational systems, Geographic Information System (GIS) and bioinformatics, respectively. The practical activity described in this paper is included in the third block, and it is scheduled for two non‐consecutive days. The number of students enrolled in this subject per year is approximately 160, who are divided into eight groups of 20 students each for this practical lesson.

In this work, an active learning approach has been implemented within the framework of the UMA Smart‐Campus (SmartUMA) to make learning more attractive and efficient. SmartUMA aims to become a global reference in environmental sustainability by implementing the campus as a Smart City,^3^ capable of supporting fully efficient infrastructures, management, research support, and learning activities.^4^ This innovative approach intends to fulfill the educational needs of the students by enhancing the learning culture outside the classroom. The application of the Smart‐Campus concept, together with the use of active learning strategies, generates enriching, motivating, and highly profitable training experiences which are mainly focused on the development of professional skills.

Active methods are teaching and learning techniques that involve students in their learning process, encouraging them to participate, think, create, and investigate more actively and critically. These strategies are designed to promote deeper understanding, retention of information, problem solving, and the development of autonomy.^5^, ^6^ This methodology can be tailored to individual needs and allows for personalized and adaptable educational experiences. The active learning methods Problem Based Learning (PBL) and Game Based Learning (GBL) have been chosen as training strategies in this intervention proposal. In PBL, students work together to solve complex and authentic problems. They seek out relevant information, analyze data, and apply their knowledge to propose well‐reasoned solutions.^7^ GBL combines game‐like elements with educational activities to engage students and enhance the acquisition of non‐formal and formal knowledge. This makes learning more dynamic, challenging, and interactive.^8^ In both methods, teachers act as facilitators, guiding and supporting students throughout the learning process rather than delivering content through traditional lectures.

**FIGURE 1:**
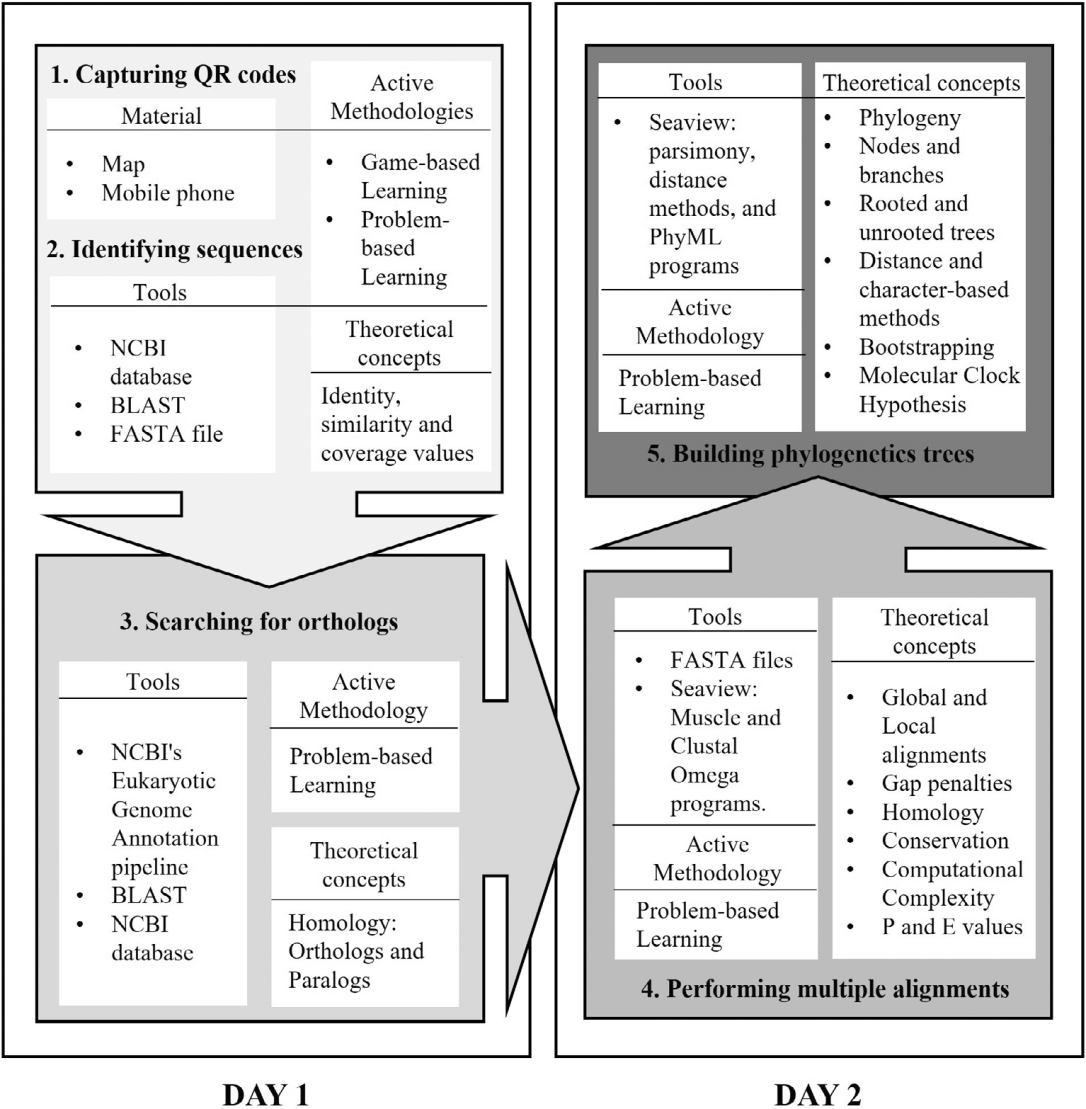
Workflow for the proposed activity.

The proposed intervention follows these learning strategies so that students will participate in a kind of gymkhana to collect 8 QR‐encoded protein sequences, which are scattered around the UMA Botanical Garden, using a QR reader and a map of the sampling area. This game provides the starting material for a bioinformatics study that consists of solving two main problems: the identification of the sequences (protein and gene names and species to which they belong) and the phylogenetic analysis of the identified species. Finally, the students are expected to draw conclusions about their evolutionary relationships. During the practical sessions, they will (1) run the Basic Local Alignment Search Tool (BLAST) to identify the sequences collected, (2) search for orthologs to learn the organization and content of molecular databases, (3) perform sequence alignments and phylogenetic tree building to learn the use of these bioinformatic applications, (4) understand the phylogeny using the appropriate terminology, (5) evaluate the suitability of using nucleic acid and/or protein sequences instead of morphological data for phylogenetic analyses, (6) learn the meaning and importance of an evolutionary clock and the qualities it should have (Figure 1). In addition, students will write a report to present the results and conclusions of the study. On the one hand, this task aims to improve their academic writing style. On the other hand, undergraduates will learn to discuss experimental results in the context of molecular evolution.

The practical lesson proposed in this work was taught to first year Biology students in the academic years 2020/21 and 2021/22.

## 2 MATERIALS AND METHODS

### 2.1 QR code generation

QR codes for a text were generated using the free online tool “qrcodemonkey” that is available at https://www.qrcode-monkey.com/#text. First, the protein sequence was copied into the “Your Text” field of the ENTER CONTENT table A different shape of eyeball and eye frame was selected for each team by clicking on the CUSTOMIZE DESIGN menu. Next, the coding was created by pressing the green “Create QR Code” link and could be downloaded in .png, .svg, .pdf or .eps format.

### 2.2 Molecular databases

The National Center for Biotechnology Information (NCBI) (https://www.ncbi.nlm.nih.gov/) provides a large collection of online resources for gene and protein information.^9^ Of these, GenBank and Protein were used primarily to collect sequence data in this work. Other molecular databases, such as European Bioinformatics Institute (EBI) or the Universal Protein Resource (Uniprot), can also be consulted as they provide similar data but with different organization. The NCBI Reference Sequence Database (https://www.ncbi.nlm.nih.gov/refseq/) was used to search for DNA, RNA, and protein reference sequences. The NCBI Eukaryotic Genome Annotation Pipeline is a bioinformatics tool that integrates the Nucleotide, Protein, BLAST, Gene and Genome Data Viewer databases to calculate orthologous sequences. To find orthologs, the gene name was entered into the NCBI search engine and the orthologs option was selected from the menu of suggestions (https://www.ncbi.nlm.nih.gov/search/).

### 2.3 Basic local alignment search tool (BLAST)

The BLAST searches for sequences (subject) that are similar to a given one (query) in a molecular database and calculates the statistical significance between their sequence matches.^10^ The tool is available at https://blast.ncbi.nlm.nih.gov/Blast.cgi. Protein BLAST (also called blastp) was used to identify query proteins as follows: (1) the blastp page was accessed by clicking on the “Protein BLAST” link, (2) the query sequence was pasted in, (3) the non‐redundant protein sequence (nr) database and the blastp algorithm were selected, from the “Choose Search Set” and “Program Select” boxes, respectively, and (4) BLAST is launched by clicking on the “BLAST” link.^11^

### 2.4 Sequence alignments and phylogenetics performance

The Seaview program was used to perform multiple sequence alignments and molecular phylogenies.^12^ This multiplatform software can be downloaded from the Rhône‐Alpes Bioinformatics Center (PRABI)‐Doua page: http://doua.prabi.fr/software/seaview. The user's manual that contains the program description and instructions is available online.^13^ Several file formats of sequences, such as FASTA, and phylogenetic diagrams can be read and written by this driver. The FASTA format is a way of presenting molecular sequences using a specific single‐letter code of DNA or protein. It begins with a single line that starts with a greater‐than symbol (“>”) followed by a description (gene or protein name, database identifier, taxon, etc.). The following lines include the sequence in plain text. For multiple sequence alignments, Seaview runs both the Muscle and Clustal Omega programs.^14^, ^15^ Phylogenetic parsimony and distance trees were computed by PHYLIP's dnapars/protpars and NJ or BioNJ algorithms, respectively. An additional maximum likelihood tree construction method could be run using the external program PhyML 3.1. Seaview also includes the Transfer Bootstrap Expectation method for calculating the statistical bootstrap support of phylogenetic trees.^16^

### 2.5 Implementation

The approximately 160 students who attended each year were divided into eight groups. Training took place from Monday to Thursday for 2 weeks, in morning and afternoon shifts. Each group of about 20 participants, attended the same day and shift each week. Finally, four teams of the same number of participants (named after the continents they represented: Asia, America, Africa, and Oceania) were formed within each group. Each team was assigned a specific protein that was encoded by a symbol (eye frame and eyeball shapes: “squircle”, square, leaf, and circle, respectively).

Each practical session included the following learning steps: (1) a short introduction by the teacher explaining the objectives and the procedure of the activity; it is worth mentioning that the theoretical issues that the students needed to know were taught during the course lectures; (2) the performance of the work required by the students in a computer science classroom; and (3) the submission of an assignment by the students through the virtual campus for each day. Although students worked and discussed together, the assignment had to be completed and presented individually.

At the end of activity, students were required to produce a final report using the following teacher's recommendations for results and discussion:

#### 2.5.1 Identification of protein and gene sequences

It was recommended that information on the location, structure and function of genes and proteins be gathered using the molecular databases that the teacher had previously explained. For this activity, one nuclear and three mitochondrial genes were chosen: succinate dehydrogenase complex flavoprotein subunit A (SDHA), ATP synthase 6 (MT‐ATP6), cytochrome b (MT‐CYTB) and NADH dehydrogenase 4 (MT‐ND4) for the Asia, America, Africa and Oceania teams, respectively.

#### 2.5.2 Search for orthologous sequences

It was necessary to describe the process for searching for orthologs and the selection criteria for distinguishing them from paralogs. Orthologs and paralogs diverge by speciation and duplication, respectively.^17^ It is essential that students have a good understanding of these key concepts because of their relevance in contexts such as phylogenetic tree interpretation and protein function annotation.

#### 2.5.3 Performing multiple alignments and phylogenetic tree construction

Two sequence alignments (Clustal Omega and Muscle) and two cladograms per alignment (parsimony and distance methods) were performed on DNA and protein sequences, separately. This resulted in a total of 4 alignments and 8 phylogenetic diagrams. After displaying the phylogeny, students were able to infer the conservation of genetic sequences between species. They could also explain (1) how the results differed depending on the methods and programs used, (2) how they were different from or similar to those hypothesized on the basis of morphological, anatomical or behavioral features rather than those based on molecular sequences. From this discussion it could be concluded whether or not the sequence (protein or DNA) under consideration was a good candidate for a molecular clock.

### 2.6 Student assessment system

An evaluation form was designed to assess the student's final report (Table 1). This report had to: be written as a scientific paper of a maximum of 10 pages, following specific format, structure and content requirements (Doc.1, Supplementary material); include a title, abstract, introduction, objectives, methodology, results, discussion and references; and be uploaded to the virtual campus in pdf format within 2 weeks. The report was divided into different sections, each of which was scored independently using a Likert‐type scale: poor (0), somewhat poor (0.25), somewhat good (0.5), good (0.75) and very good (1). The overall qualification of the report was calculated using a weighted average method, ensuring that the score of each section contributes proportionally to the final grade, which is up to 10, according to the weight assigned to it. For each score range, below 5, 5 to 7, 7 to 9 and above 9, the achievement levels assigned are below basic, basic, proficient and advanced. Below Basic indicates inadequate knowledge and skills, not meeting minimum standards and requiring significant support. Basic reflects limited understanding and basic skills, meeting minimum standards but requiring further development. Proficient demonstrates notable knowledge and competent skills, meeting expected standards and applying knowledge. Advanced demonstrates solid knowledge and advanced skills, exceeding standards and applying knowledge effectively. The assessment focused on three key aspects: the development of computer skills to manage sequence databases and sequence analysis software, the understanding of theoretical concepts through their practical application in real data interpretation, and the overall quality of the submitted work based on written communication abilities using technical and scientific terminology.

**TABLE 1:** Student assessment system.

Likert‐Type Scale	Poor	Somewhat poor	Somewhat good	Good	Very good	Score	Weighted score
**1. Format requirements**						Up to 6	Up to 1
1.1 Font and size	0	0.25	0.5	0.75	1		
1.2 Margins and justified text	0	0.25	0.5	0.75	1		
1.3 Scientific names	0	0.25	0.5	0.75	1		
1.4 Figures and tables	0	0.25	0.5	0.75	1		
1.5 References	0	0.25	0.5	0.75	1		
1.6 Documents delivery	0	0.25	0.5	0.75	1		
**2. Introduction**	0	0.25	0.5	0.75	1	Up to 1	Up to 0.5
**3. Abstract**	0	0.25	0.5	0.75	1		
**4. Objectives**	0	0.25	0.5	0.75	1		
**5. Material and methods**						Up to 2	Up to 2
5.1 Material	0	0.25	0.5	0.75	1		
5.2 Methods	0	0.25	0.5	0.75	1		
**6. Results**						Up to 4	Up to 2.5
6.1 Sequences identification	0	0.25	0.5	0.75	1		
6.2 Search for orthologs	0	0.25	0.5	0.75	1		
6.3 Alignments	0	0.25	0.5	0.75	1		
6.4 Phylogenetics trees	0	0.25	0.5	0.75	1		
**7. Discussion and conclusions**						Up to 4	Up to 2.5
7.1 Sequences identification	0	0.25	0.5	0.75	1		
7.2 Search of orthologs	0	0.25	0.5	0.75	1		
7.3 Alignments	0	0.25	0.5	0.75	1		
7.4 Phylogenetics trees	0	0.25	0.5	0.75	1		
**8. Bibliography**	0	0.25	0.5	0.75	1	Up to 1	Up to 0.5
Overall qualification (weighted average score)							**Up to 10**

### 2.7 Student survey

A brief questionnaire to evaluate the student feedback was made available on the UMA virtual campus after the activity and its evaluation had been completed. This survey (Figure S1) was voluntary and anonymous, and all students were encouraged to complete it in order to improve both the process and the assessment system.

## 3 RESULTS AND DISCUSSION

In the academic years 2020/21 and 2021/22, a total of 244 students completed the activity and submitted the final report. Table S1 shows the initial number of participants (320), of whom 83% and 78% performed the activity in their respective courses. This may be because the genetics block is the last module to be taught and approximately 20% of the students had already withdrawn. Of the remaining 80%, about 87% completed all the tasks.

### 3.1 Day 1. Searching for information in molecular databases

**FIGURE 2:**
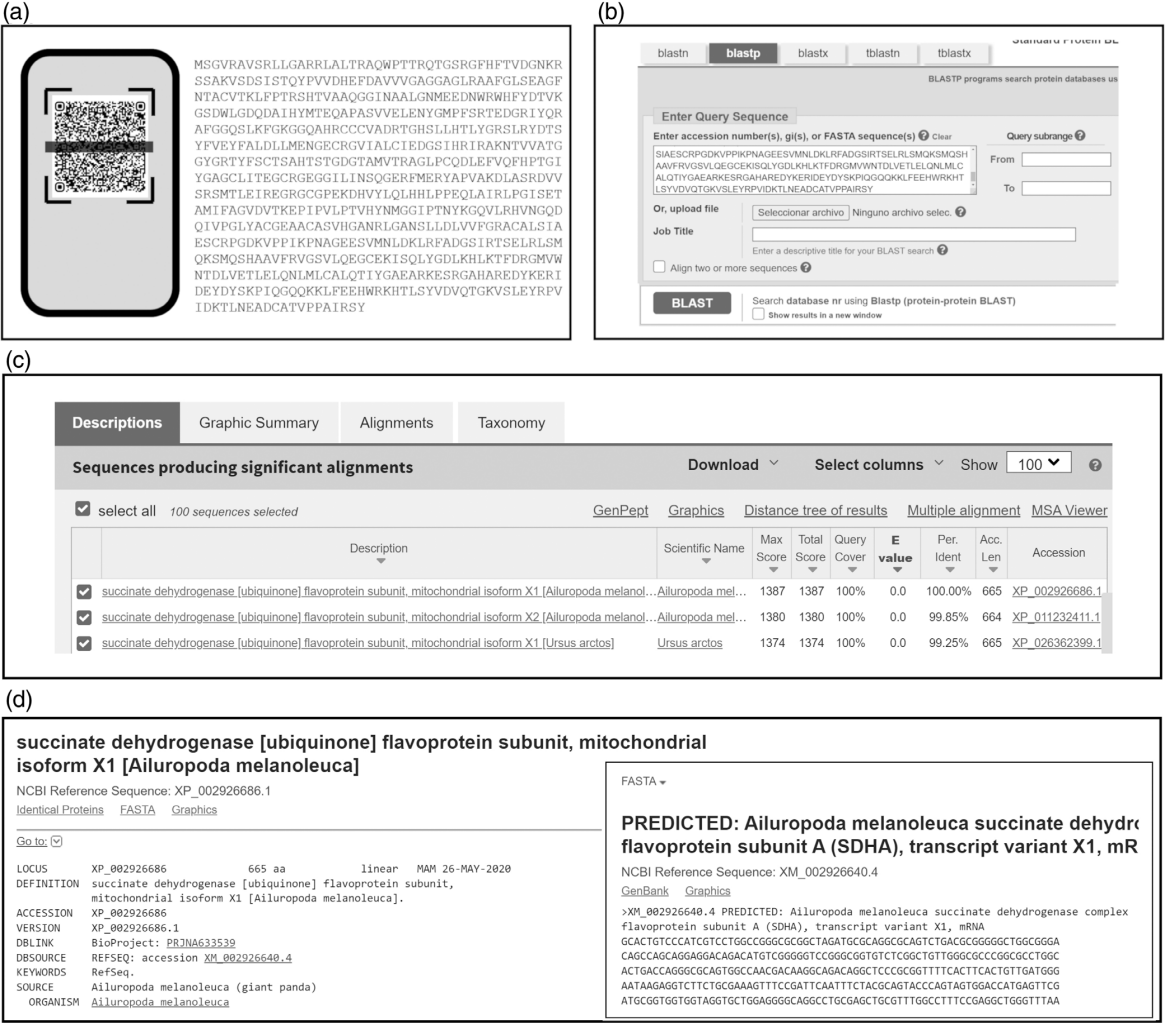
Sequence identification process using BLAST.

During the first half hour, each team looked for 8 QR codes, each identified with its corresponding symbol, around the UMA Botanical Garden with the help of a map. Once the students had scanned all the QR codes (Figure 2a) (Figure S2) with their mobile phones, they returned to the classroom where they carried out the following two steps:

#### 3.1.1 Identification of the captured sequences using BLAST

The students connected to https://blast.ncbi.nlm.nih.gov/Blast.cgi and clicked on “Protein BLAST” (blastp). They then copied and pasted the protein sequences from the QR code into the “Enter query sequence” field and clicked on the BLAST link (Figure 2b). Many alignments were generated between the query and subject sequences. The results were sorted by certain factors such as query coverage and percent of identity. To determine the identity of the query sequence, both parameters had to be 100% (Figure 2c). If there was more than one match, students had to select the one belonging to the reference sequence collection (RefSeq), as it provides a comprehensive, integrated, non‐redundant and well‐annotated set of sequences. After identification, the students searched for the corresponding nucleotide sequences by accessing the appropriate database or using the “accession” link that appears on the BLAST results page (Figure 2d).

Finally, they produced two text files in FASTA format containing the amino acid and nucleotide sequences, respectively. The sequence descriptors were the scientific names of the species (Doc.2–3, Supplementary material), and the files were named with the protein and gene names.

#### 3.1.2 Finding orthologous sequences

Once the students had established the identity of the sequences and the name of the corresponding species, this set of sequences was completed by adding 16 more, eight of each type of sequence (DNA and protein), from other animal species, for a more accurate, reliable, and reproducible phylogenetic analysis. Most of the samples had to be vertebrates and at least two of them had to be invertebrates, which served as an outgroup (the species most distantly related to the rest).

The search for orthologs could be performed using tools such as the NCBI keyword search engine, the BLAST filtering by species, and the NCBI Eukaryotic Genome Annotation pipeline, which calculates orthologous, gene groups for the NCBI Gene dataset. These orthologous sequences were added to the corresponding previously generated text files in the same format.

These two text files had to be uploaded to the virtual campus at the end of the session. Students who did not complete the activity in class had 24 h to submit the assignment.

### 3.2 Day 2. Sequence alignment and molecular phylogeny

During this session, students performed a graphical multiple sequence alignment using the previously obtained orthologs and built phylogenetic trees for the molecular evolutionary study of vertebrate species. They took screenshots of the results of both analyses to create a draft of their required scientific report.

#### 3.2.1 Performing multiple alignments using the 16 orthologous sequences

**FIGURE 3:**
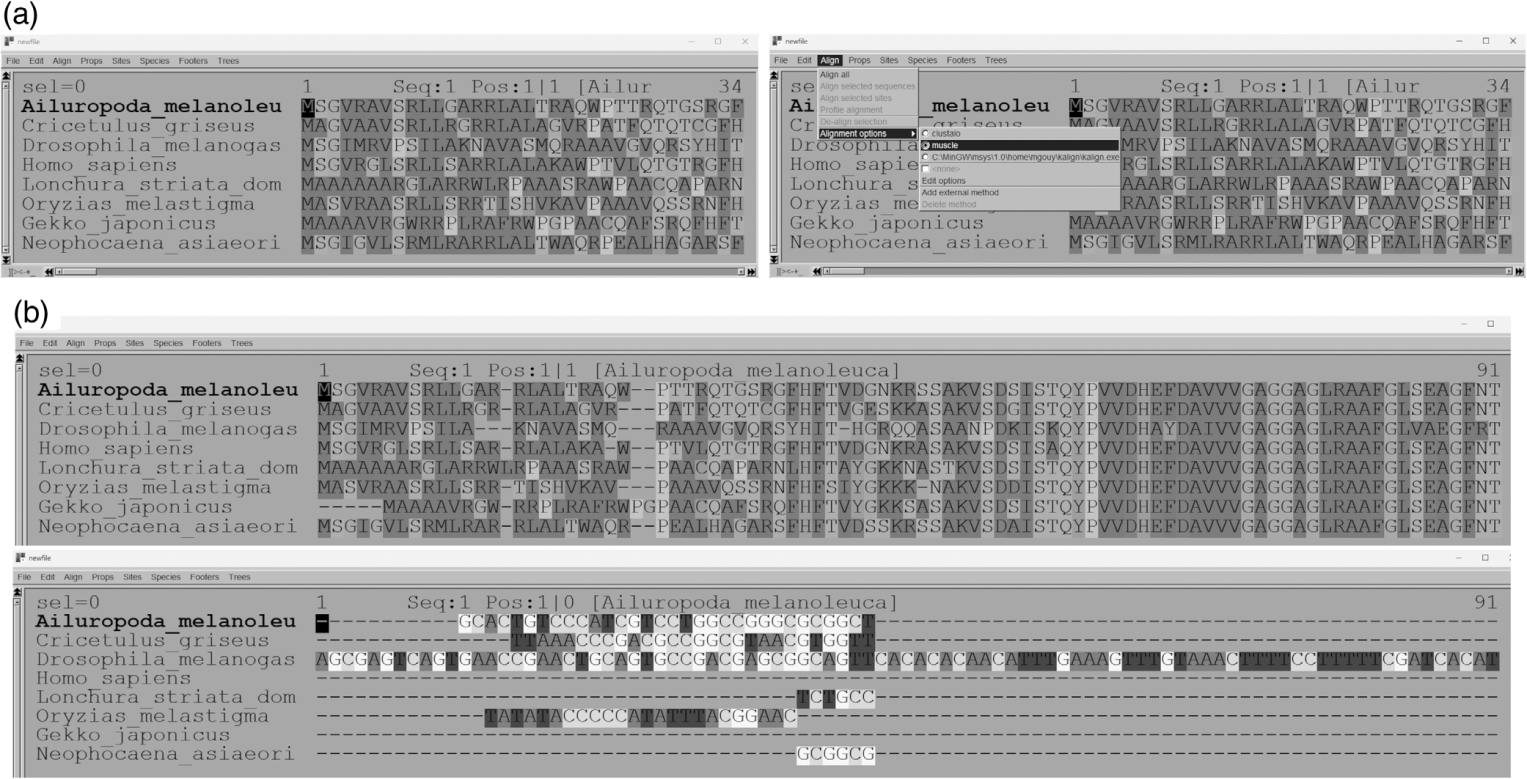
Performing multiple alignments.

Two separate windows of the Seaview program were used to open the nucleotide and amino acid sequence lists. This software includes the Muscle and Clustal Omega programs (Figure 3a). Both alignment options were performed on each set of sequences (Figure 3b). Students compared their results and wrote down the differences they observed if applicable.

#### 3.2.2 Phylogenetic tree construction

Seaview also includes three programs for calculating and drawing phylogenetic trees: parsimony, distance methods, and PhyML. Because PhyML uses an external program to compute trees, it takes longer than the others. Therefore, students practised only parsimony and distance methods.

**FIGURE 4:**
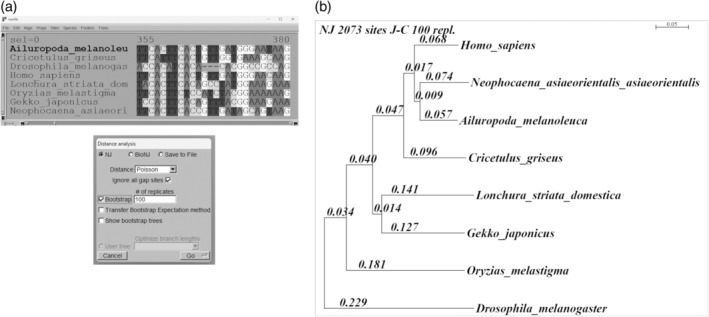
Building distance phylogenetic trees.

First, they clicked on the “trees” menu and then on the “distance methods” option. A new window appeared, and the tree‐building NJ (neighbor‐joining) algorithm was selected, as well as the bootstrap choice with 100 replicates (Figure 4a). The resulting tree was modified as follows (Figure 4b): 18‐point font size was selected by clicking on the “font” menu; the tree was re‐rooted with the outgroup if necessary; the operational taxonomic units (OTUs) were arranged according to the order of the reference phylogeny^18^ (Figure S3) using the “swap” function; “full” had to be clicked to select the display of branch lengths (Br length) or bootstrap support (Br support).

As a result, four trees were generated using the Clustal Omega and Muscle programs, both with protein and nucleotide sequences.

**FIGURE 5:**
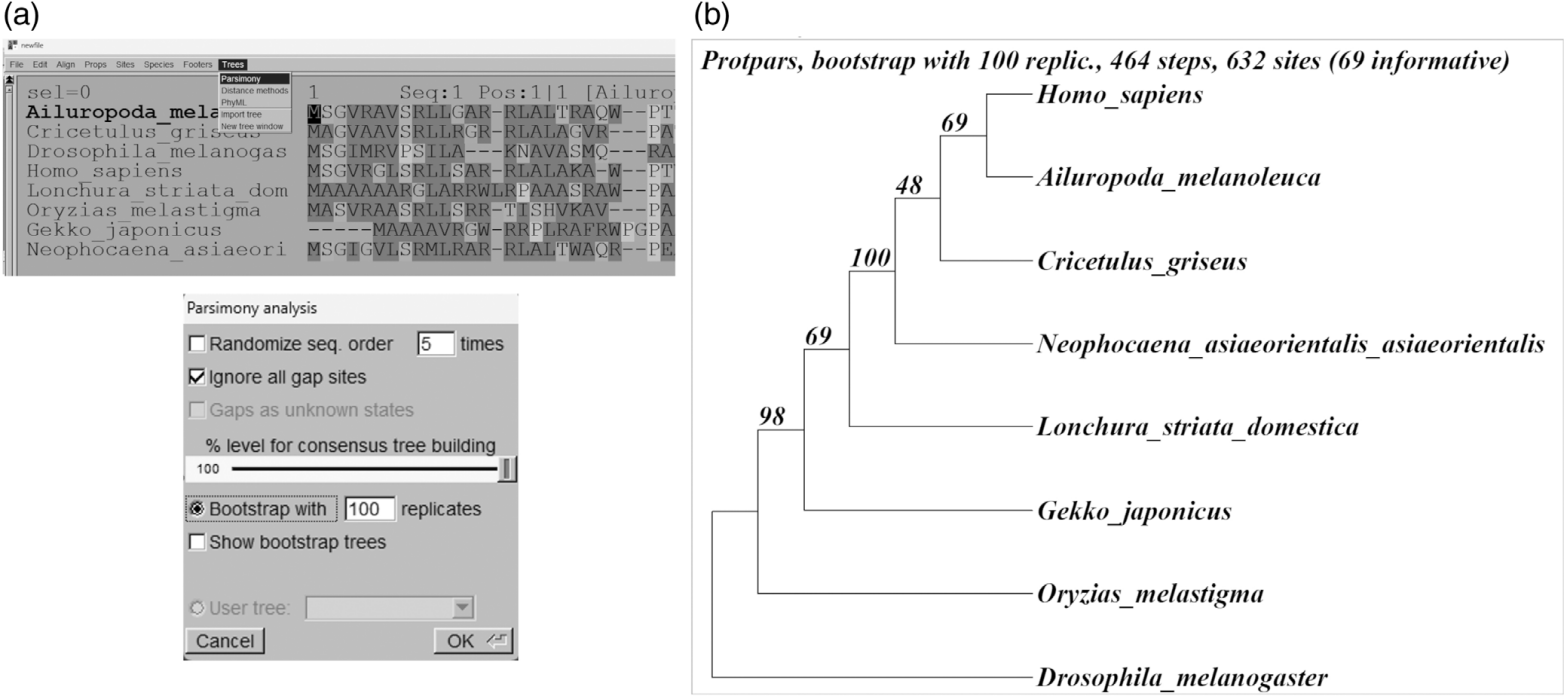
Computing parsimony trees.

Finally, the same procedure was used to generate four parsimony trees (Figure 5a). Although the “circular” tree visualization is the most appropriate for this method, it was presented as a “square” cladogram for comparison with the distance tree, for which it must be re‐rooted with the invertebrate species (Figure 5b).

## 4 LESSON LEARNING OUTCOMES

### 4.1 Student feedback

**FIGURE 6:**
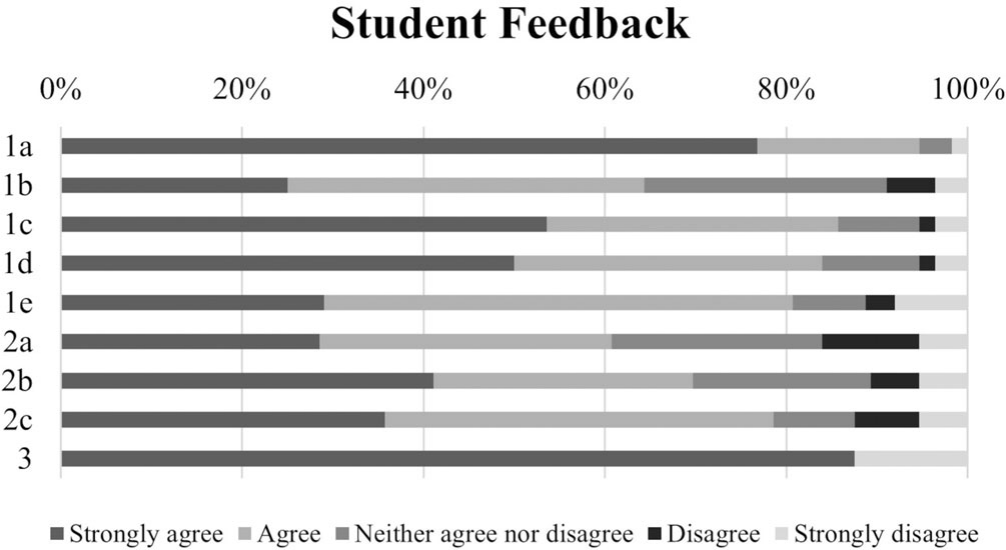
Student feedback.

Students were invited to complete a 10‐question anonymous online survey to find out their level of satisfaction with the lesson and their opinion of the importance and benefits of this practice both in the understanding of theoretical concepts and in developing interpersonal and information management skills. The survey outcomes are shown in Figure 6.

The results of the survey indicate a positive response to the Computer Science practical lesson, with high percentages of agreement on various aspects: 94.6% found the lesson important and 85.7% and 83.9% found it useful for acquiring and consolidating subject knowledge, respectively. In addition, 89.3% felt that the lessons were effective in helping them to learn how to find, use and manage information, and a substantial majority of 87.5% considered the assessment system to be appropriate. However, there is scope for improvement in other aspects: 64.3% felt it encouraged student interaction and the satisfaction with the quality of teaching was acceptable, with 60.7% satisfied with the explanations, 69.6% with the resolution of doubts and 78.6% with the resources available such as protocols, report guides and tutorials.

Although these findings indicate an overall positive student experience and perception of the efficacy of the teaching methods and support systems in place for the subject, the comments left by students indicate some relevant areas for improvement that can be summarized as follows:

1. The theoretical content provided was initially inadequate, which later led to confusion.
2. During the practical sessions there was a lack of clarity about the tasks to be completed.
3. Students emphasized the importance of practical sessions over theoretical lectures, which often consisted of slide presentations.
4. They requested more detailed explanations, especially when using software programs and databases.

### 4.2 Student assessment

**FIGURE 7:**
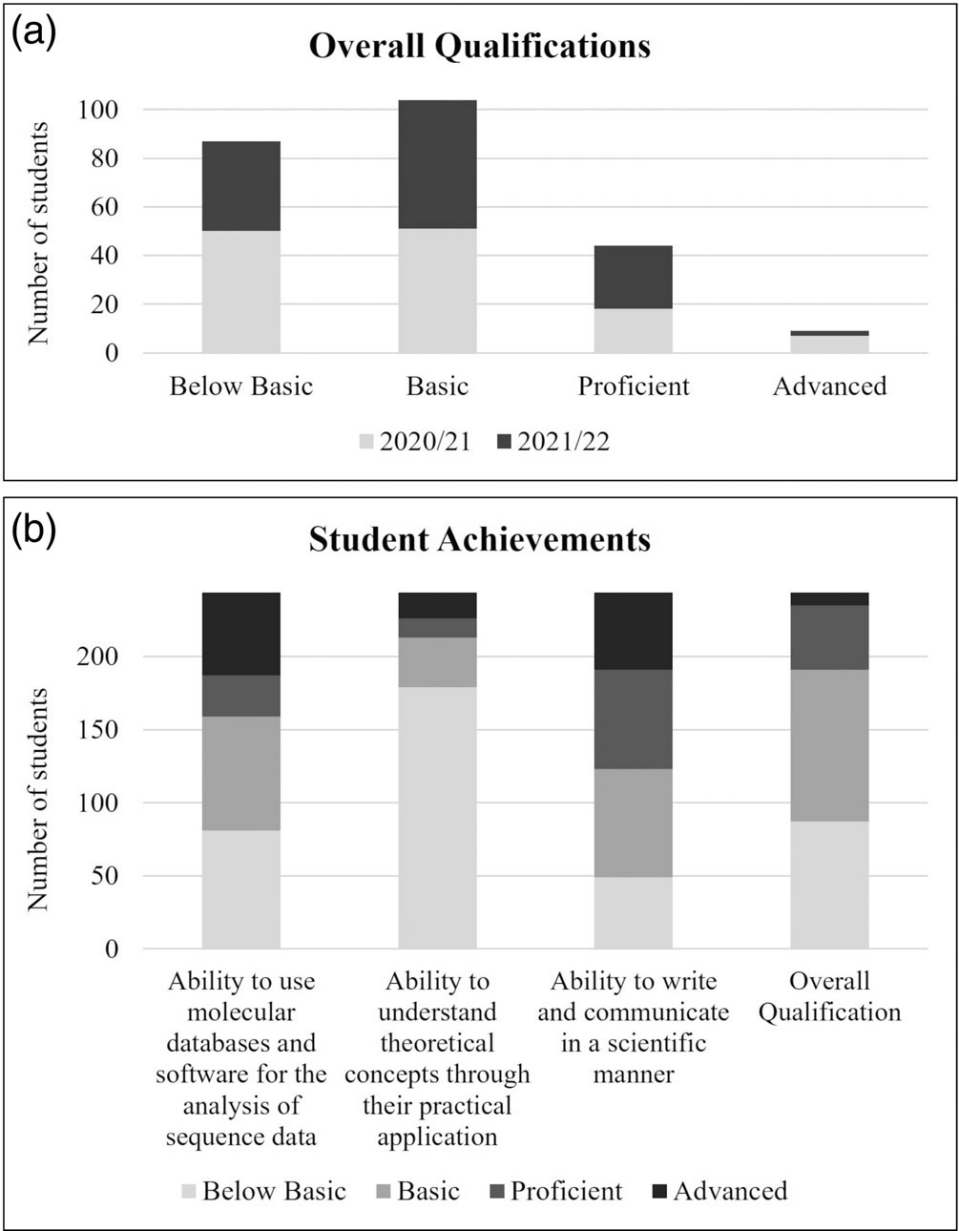
Student learning outcomes.

Student performance was assessed according to the criteria set out in the evaluation form (Table 1). This system was also designed to ensure fairness, consistency and objectivity in the qualifications awarded. The results of the assessment are shown in Figure 7.

The overall qualification demonstrated the students' understanding of the subject matter, the quality of their analysis, the coherence of their arguments and their ability to communicate complex ideas clearly and accurately. In this sense, this didactic proposal proved to be effective in most cases, with 64.3% (60.3% for 2020/21 and 68.6% for 2021/22) of students demonstrating knowledge and skills that met the set standards, compared to 35.7% (39.7% for 2020/21 and 31.4% for 2021/22) who did not meet the minimum requirements and needed significant support (Figure 7a). A chi‐squared test was performed to examine the association between proficiency levels and academic years (2020/21 and 2021/22). The results showed a chi‐squared value of χ^2^ ≈ 5.96 with 3 ° of freedom, and a *p*‐value of 0.1137. Since the *p*‐value is greater than the significance level of 0.05, there were no significant differences between the qualifications of the different academic years.

The “Results” section of the assessment form was specifically designed to assess students' ability to use molecular databases and software to analyze sequence data. In this respect, two‐thirds of the students showed skills that ranged from basic to competent. The “Discussion” section was used to demonstrate their ability to apply theoretical concepts to real‐world scenarios, such as the context of this didactic activity: the phylogeny and evolution of vertebrates. These were the aspects where students showed the greatest difficulty, with most failing to meet the minimum standards (73.4% compared to 26.6%). As the other sections of the form reflect the overall quality of writing, including the appropriate application of technical and scientific terminology, they were used to assess students' ability to write and communicate in a scientific manner. As a result, 80% of the students were able to present their work in an appropriate style, with basic (30%), competent (28%) and advanced (21%) levels (Figure 7b).

Following the evaluation, the revised report and assessment form were made available to students, together with a list of common errors (Doc. 4, Supplementary Material), with the aim of enhancing the learning experience, reinforcing the basic concepts of molecular phylogeny, and improving the scientific rigor and clarity of the student reports.

## 5 CONCLUSIONS

The evaluation and feedback mechanisms used in this work have provided valuable insights into both student performance and satisfaction. It has also allowed to identify areas of improvement for future interventions.

Students highly valued the practical sessions highly and emphasized their importance over traditional theoretical lectures, which were initially perceived as lacking in content. This suggests the need for a balanced integration of theoretical content and practical applications to provide a solid foundation, reduce confusion and reinforce key concepts at an early stage. Clarity of task requirements during the practical sessions emerged as a significant concern. Clearer instructions and expectations could improve student engagement and productivity during these sessions. There was also a strong demand for more detailed explanations, particularly when using software programs and databases. It would therefore be necessary to develop more comprehensive resources and tutorials to address the specific needs of students for detailed guidance and to provide effective feedback to link the information taught with the application of what has been learned.^19^

The Bioinformatics subject provides an introduction to sequence analysis for first‐year students who are encountering this theoretical and procedural content for the first time. Considering this and the learning outcomes, it would also be advisable to simplify the conceptual content in order to facilitate comprehension. In this context, understanding students' prior knowledge and what they are curious about would be a relevant aspect in the design of training instructions, as prior knowledge has a positive impact on the acquisition of new information, and motivation to learn seems to play a role in facilitating this association.^20^ Similarly, a regular feedback loop in the light of continuous student input would be necessary to monitor and adjust teaching methods, assessment criteria and resources.

By implementing these improvements, this hands‐on molecular phylogeny activity will provide a valuable opportunity for students to develop their ability to search for information in molecular databases and to analyze phylogenetic relationships using DNA and protein sequences. Students will also develop their ability to identify and solve problems that may arise in an experimental situation, and to interpret and draw conclusions from the results obtained.

## AUTHOR CONTRIBUTIONS

**Inmaculada Ortiz Martín**: conceptualization; methodology; resources; writing‐original draft; writing‐review & editing; visualization; project administration. **Ángel Del Espino Pérez**: conceptualization; methodology; resources; reviewing‐manuscript. **Estefanía García Luque**: conceptualization; visualization; reviewing‐manuscript. **Enrique Viguera Mínguez**: conceptualization; methodology; reviewing‐manuscript; project administration.

## CONFLICT OF INTEREST STATEMENT

Authors have no conflict of interest to declare.

## Supporting information


**Table S1.** Study participants and their assessment results.

**Figure S1.** Student survey questions.

**Figure S2.** QR codes.

**Figure S3.** Phylogeny of vertebrates.

**Data S1. Doc.1 Supplementary material.** Format, structure and content requirements of the student report.

**Data S2. Doc.2 Supplementary material.** Protein sequences provided in this work.

**Data S3. Doc.3 Supplementary material.** DNA sequences provided in this work.

**Data S4. Doc.4 Supplementary material.** The most common errors and learning difficulties encountered by the students in the preparation of the final report.

## Data Availability

Data sharing not applicable – no new data generated, or the article describes entirely theoretical research.

## References

[1] Mayr E . Populations, species, and evolution. New York: Belknap Harvard; 1970.

[2] Zuckerkandl E , Pauling L . Molecules as documents of evolutionary history. J Theor Biol. 1965;8(2):357–366. 10.1016/0022-5193(65)90083-4 5876245

[3] Schaffers H , Komninos N , Pallot M , Trousse B , Nilsson M , Oliveira A . Smart Cities and the Future Internet: Towards Cooperation Frameworks for Open Innovation. In: Domingue J, et al. The Future Internet. FIA 2011. Lecture Notes in Computer Science, vol 6656. Springer, Berlin, Heidelberg. 2011.

[4] Fortes S , Santoyo‐Ramón JA , Palacios D , Baena E , Mora‐García R , Medina M , et al. The campus as a Smart City: University of Málaga Environmental, learning, and research approaches. Sensors. 2019;19(6):1349. 10.3390/s19061349 30889886 PMC6471123

[5] Sivan A , Wong Leung R , Woon C‐C , Kember D . An implementation of active learning and its effect on the quality of student learning. Innov Educ Train Int. 2000;37(4):381–389. 10.1080/135580000750052991

[6] Freeman S , Eddy SL , McDonough M , Smith MK , Okoroafor N , Jordt H , et al. Active learning increases student performance in science, engineering, and mathematics. Proc Natl Acad Sci USA. 2014;111(23):8410–8415. 10.1073/pnas.1319030111 24821756 PMC4060654

[7] Savery JR . Overview of problem‐based learning: definitions and distinctions. Interdis J Probl Bas Learn. 2006;1(1):9‐20. 10.7771/1541-5015.1002

[8] Lei H , Chiu MM , Wang D , Wang C , Xie T . Effects of game‐based learning on Students' achievement in science: a meta‐analysis. J Educ Comput Res. 2022;60(6):1373–1398. 10.1177/07356331211064543

[9] National Center for Biotechnology Information (US) . NCBI Help Manual [Internet]. Bethesda (MD): National Center for Biotechnology Information (US). 2005. https://www.ncbi.nlm.nih.gov/books/NBK3831/

[10] Madden T . The BLAST Sequence Analysis Tool. In: The NCBI Handbook [Internet]. 2nd edition. Bethesda: National Center for Biotechnology Information . 2013. https://www.ncbi.nlm.nih.gov/books/NBK153387/

[11] Bergman NH , editor. Comparative genomics: volumes 1 and 2. Totowa (NJ): Humana Press; 2007. 21250292

[12] Gouy M , Guindon S , Gascuel O . SeaView version 4: a multiplatform graphical user interface for sequence alignment and phylogenetic tree building. Mol Biol Evol. 2010;27(2):221–224. 10.1093/molbev/msp259 19854763

[13] Gouy M . On‐line help document. 2020 http://doua.prabi.fr/software/seaview_data/seaview

[14] Edgar RC . MUSCLE: multiple sequence alignment with high accuracy and high throughput. Nucleic Acids Res. 2004;32(5):1792–1797. 10.1093/nar/gkh340 15034147 PMC390337

[15] Sievers F , Wilm A , Dineen D , Gibson TJ , Karplus K , Li W , et al. Fast, scalable generation of high‐quality protein multiple sequence alignments using Clustal omega. Mol Syst Biol. 2011;7:539. 10.1038/msb.2011.75 21988835 PMC3261699

[16] Lemoine F , Domelevo Entfellner JB , Wilkinson E , Correia D , Dávila Felipe M , De Oliveira T , et al. Renewing Felsenstein's phylogenetic bootstrap in the era of big data. Nature. 2018;556(7702):452–456. 10.1038/s41586-018-0043-0 29670290 PMC6030568

[17] Koonin EV . Orthologs, paralogs, and evolutionary genomics. Annu Rev Genet. 2005;39:309–338. 10.1146/annurev.genet.39.073003.114725 16285863

[18] Benton MJ . The quality of the fossil record of vertebrates. In: Donovan SK , Paul CRC , editors. The adequacy of the fossil record. New York: Wiley; 1998.

[19] Al Abadi H , Mainali B , Lumantarna E . Teaching advancement with phased assessments and looped feedback (PALF) model. Eur J Eng Educ. 2022;47(6):1229–1242. 10.1080/03043797.2022.2145937

[20] Witherby AE , Carpenter SK . The rich‐get‐richer effect: prior knowledge predicts new learning of domain‐relevant information. J Exp Psychol Learn Mem Cogn. 2022;48(4):483–498. 10.1037/xlm0000996 33539165

