# Supplementary material for: Using active learning methodologies to teach sequence analysis and molecular phylogeny
Source: Biochem Mol Biol Educ. 2024 Oct 14;53(1):21–32. doi: 10.1002/bmb.21861 (PMC11752413; doi:10.1002/bmb.21861)
Supplement: Supplementary file 1 — Table S1. Study participants and their assessment results. [file BMB-53-21-s008.docx]

| **Number of students who…** | **Academic Year** | | | | | | | |
| --- | --- | --- | --- | --- | --- | --- | --- | --- |
|  | **2020/21** | | | | **2021/22** | | | |
| Were enrolled in the subject | 169 | | | | 151 | | | |
| Completed the activity | 139 | | | | 139 | | | |
| Submitted the final report | 126 | | | | 118 | | | |
| **Number of students who demonstrated the ability…** | **Achievement levels** | | | | | | | |
|  | Below basic | | Basic | | Proficient | | Advanced | |
| To use molecular databases and software to analyze sequence data | **20/21** | **21/22** | **20/21** | **21/22** | **20/21** | **21/22** | **20/21** | **21/22** |
|  | 41 | 40 | 40 | 38 | 19 | 9 | 26 | 31 |
| To understand theoretical concepts through their practical application | **20/21** | **21/22** | **20/21** | **21/22** | **20/21** | **21/22** | **20/21** | **21/22** |
|  | 84 | 95 | 20 | 14 | 8 | 5 | 14 | 4 |
| To write and communicate in a scientific manner | **20/21** | **21/22** | **20/21** | **21/22** | **20/21** | **21/22** | **20/21** | **21/22** |
|  | 39 | 10 | 38 | 36 | 32 | 26 | 17 | 36 |
| Overall qualification | **20/21** | **21/22** | **20/21** | **21/22** | **20/21** | **21/22** | **20/21** | **21/22** |
|  | 50 | 37 | 51 | 53 | 18 | 26 | 7 | 2 |
