# Supplementary material for: Using active learning methodologies to teach sequence analysis and molecular phylogeny
Source: Biochem Mol Biol Educ. 2024 Oct 14;53(1):21–32. doi: 10.1002/bmb.21861 (PMC11752413; doi:10.1002/bmb.21861)
Supplement: Supplementary file 5 — Data S1. Doc.1 Supplementary material. Format, structure and content requirements of the student report. [file BMB-53-21-s001.docx]

**Format, Structure and content requirements of the student report**

The findings of the two-day experimental class will be documented in a report that must be prepared in accordance with the standards set forth in this document. The document should not exceed ten pages in length. The paper must include the following sections: abstract, introduction, objectives, methodology, results, discussion and bibliography. The document must be submitted in PDF format.

**Format requirements**

All printed material, including text, illustrations and diagrams, must be confined to the designated print area. The dimensions of all pages must be A4. The primary title, located on the initial page, must be centred and presented in 14-point bold Times New Roman font. The initial letter of nouns, pronouns, verbs, adjectives, and adverbs are to be capitalised. The name and affiliations of the author must be centred below the title and presented in 12-point italic font. It is preferable to include the email address in the same format. The font size must be 11-point Times New Roman with a single line spacing. It is imperative that no space be included before or after the paragraph. All paragraphs must commence with an indentation of 0.4 cm in the first line. It is required that a blank line be inserted between paragraphs. It is not permissible to include headers or footers on any pages, nor should any pages be numbered. The initial level of titles must be numbered in a 12-point bold font, namely Times New Roman. It is required that a blank line be inserted before and after each paragraph. A period should be used to separate the number from the title. The second and third-level titles must adhere to the same formatting conventions as the first-order title, but with a font size of 11 points. The third-level title should be presented in italics, rather than in bold. Tables and figures must be centred, with text in 10-point Times New Roman font and the title in bold. They must have independent numbers and appear below and above them, respectively. The references must be listed and numbered in 10-point Times New Roman font at the conclusion of the report. The American Psychological Association (APA) citation format must be used.

**Structure and content requirements**

**Abstract**

A brief and concise summary that provides an overview of the content and main findings of the document. Its purpose is to give readers a clear idea of what the report is about without them needing to read the entire text.

1. **Introduction**

This section provides context for the topic of the report. It is recommended to include a concise synopsis (5-10 lines) of the significance of molecular phylogeny.

1. **Objectives**

It is imperative that the objective(s) of the activity be explicitly stated. It is important to exercise caution when selecting the terminology to be used. The use of phrases such as "I searched the databases for my protein or gene" is inaccurate.

1. **Materials and Methods**

It is necessary to identify the bioinformatics tools, databases, filters, alignment analysis programs, alignment models, phylogenetic tree construction algorithms, selected species, outgroup species, etc. that have been used. It is recommended to include a table with the scientific names of the species and their corresponding common names. It is of the utmost importance to adhere to the principles of binomial nomenclature when referring to species names. These should be presented in italics, with the genus name in capitals and the species name in lower case. This convention must be observed in all textual and graphical elements, including phylogenetic trees. Failure to comply with these instructions will result in penalties.

1. **Results**

It is required that the results obtained at each stage of the process are displayed and described, with the relevant program(s) indicated. Images of both the protein and nucleotide sequence alignment, produced by the Clustal and Mucle algorithms, and the phylogenetic trees generated by both the distance and parsimony methods must be included. The species must be ordered according to the reference tree of vertebrates and the tree must be rooted with the outgroup species. The Bootstrap values used must also be indicated.

1. **Discussion and conclusions**

The following topics may be considered in the discussion of the results: What is the underlying characteristic that unifies the sampled species? Description of any difficulties or issues encountered in searching for orthologs and how they were resolved. Commentary on the degree of conservation of the gene/protein in question, as determined by the obtained alignment. It would be beneficial to ascertain whether there are any differences between the alignments depending on the method used, and to provide an explanation for these differences.

The interpretation of the trees obtained by different methods and a comparison with the reference tree based on morphological data will be undertaken. Provide a discussion of the differences observed and, if applicable, identify any taxon for which the resolution is inadequate. Was there any indication as to whether the sequences were too divergent? Describe the method that was employed to resolve this issue. Provide a detailed account of any errors encountered by the program and the methods employed to resolve them. Would the same results have been obtained if alternative orthologous species had been selected? And any other comment worth noting.

Finally, provide a detailed account of the analysis findings as the conclusions of the study.
