# Supplementary material for: Using active learning methodologies to teach sequence analysis and molecular phylogeny
Source: Biochem Mol Biol Educ. 2024 Oct 14;53(1):21–32. doi: 10.1002/bmb.21861 (PMC11752413; doi:10.1002/bmb.21861)
Supplement: Supplementary file 6 — Data S2. Doc.2 Supplementary material. Protein sequences provided in this work. [file BMB-53-21-s004.docx]

**AMERICA**

>Ambystoma_tigrinum (YP_214776.1)

MNLSFFDQFMSPIMLGIPLILLAMTIPWLLYASPTDRWLNNRLTTLQAWFLASFTKQLMLPLNIKGHKWALPLTSLMIFLITMNLLGLLPYTFTPTTQLSLNLGLAVPFWLATVLIGLRNQPTAALGHLLPEGTPTLLIPILIIIETISLFIRPLALGVRLTANLTAGHLLIQLISTAVFVLMPMMPTTAIITAIVLFLLTLLEIAVAMIQAYVFVLLLSLYLQENT

>Caiman_crocodilus (NP_112527.1)

MNTNLFDQFMIPNLMGTPLLMPALLIIPLLLLNPKNQWLSNPATTMKSWYITQITKQIMTPINKPGHMHSVTLISLLILLSFTNLLGLLPYTFTPTTQLSMNMALALPLWGMTVLIGLRTQPTTSLAHLLPEGTPTPLIPILILIETISLLIRPVALAVRLTANLTAGHLLIQLLSMATMNLWSIMPPLSLLTLTTLTLLLLLEFAVAMIQAYVFVLLLSLYLQENT

>Drosophila_melanogaster (YP_009047270.1)

MMTNLFSVFDPSAIFNFSLNWLSTFLGLLMIPSIYWLMPSRYNIMWNSILLTLHKEFKTLLGPSGHNGSTFIFISLFSLILFNNFMGLFPYIFTSTSHLTLTLSLALPLWLCFMLYGWINHTQHMFAHLVPQGTPAILMPFMVCIETISNIIRPGTLAVRLTANMIAGHLLLTLLGNTGPSMSYILVTFLLMAQIALLVLESAVAMIQSYVFAVLSTLYSSEVN

>Himatione_sanguinea (YP_009107724.1)

MNLSFFDQFSSPSFLGIPLILISMTFPALLIPSLDNRWITNRLSTLQLWFVNLVTKQLMMPLDKKGHKWALILTSLMIFLLLINLLGLLPYTFTPTTQLSMNLALAFPLWLATLLTGLRNQPSISLGHLLPEGTPTPLIPALILIETTSLLIRPLALGVRLTANLTAGHLLIQLISTATTALLPTMPAVSLLTLLVLFLLTILEVAVAMIQAYVFVLLLSLYLQENI

>Marmota_flaviventris (YP_009632419.1)

MNENLFASFITPTLMGFPIVLFIIMFPNLLFPSPTRLVNNRLVSFQQWLIQLVLKQMMTIHNPKGRTWSL

MLISLIMFIGSTNLLGLLPHSFTPTTQLSMNLGMAIPLWAGAVITGFRYKTKASLAHFLPQGTPILLIPM

LIIIETISLFIQPMALAVRLTANITAGHLLMHLIGGATLVLTSISPPTAILTFTILVLLTMLEFAVALIQ

AYVFTLLVSLYLHDNT

>Homo_sapiens (YP_003024031.1)

MNENLFASFIAPTILGLPAAVLIILFPPLLIPTSKYLINNRLITTQQWLIKLTSKQMMTMHNTKGRTWSL

MLVSLIIFIATTNLLGLLPHSFTPTTQLSMNLAMAIPLWAGTVIMGFRSKIKNALAHFLPQGTPTPLIPM

LVIIETISLLIQPMALAVRLTANITAGHLLMHLIGSATLAMSTINLPSTLIIFTILILLTILEIAVALIQ

AYVFTLLVSLYLHDNT

>Pterois_volitans (YP_009092388.1)

MTLSFFDQFMSPTYLGIPLMALALTLPWILFPTPTARWLNNRPMTLQGWFINRFTQQLLLPLNVGGHKWA

ALLASLMIFLITLNMLGLLPYTFTPTTQLSLNLGLAVPLWLATVIIGMRNQPTHALGHLLPEGTPGPLIP

ILIIIETISLFIRPLALGVRLTANLTAGHLLIQLIATAAFVLLPLMPSVAILTTTVLVLLTLLEIAVAMI

QAYVFVLLLTLYLQENV

>Ursus_americanus (NP_597960.1)

MNESLFTSFITPTMMGIPIVVLIIMFPSILFPSPSRLVNNRLVSIQQWLVRLTSKQMLSIHNHKGQTWAL

MLMSLILFIGSTNLLGLLPHSFTPTTQLSMNLGMAIPLWTGTVAIGLRYKTKASLAHFLPQGTPFPLIPM

LVIIETISLFIQPMALAVRLTANITAGHLLIHLIGGATLALTSISTITALITFTILVLLTILEFAVALIQ

AYVFTLLVSLYLHDNT

**ASIA**

>Ailuropoda_melanoleuca (XP_002926686.1)

MSGVRAVSRLLGARRLALTRAQWPTTRQTGSRGFHFTVDGNKRSSAKVSDSISTQYPVVDHEFDAVVVGAGGAGLRAAFGLSEAGFNTACVTKLFPTRSHTVAAQGGINAALGNMEEDNWRWHFYDTVKGSDWLGDQDAIHYMTEQAPASVVELENYGMPFSRTEDGRIYQRAFGGQSLKFGKGGQAHRCCCVADRTGHSLLHTLYGRSLRYDTSYFVEYFALDLLMENGECRGVIALCIEDGSIHRIRAKNTVVATGGYGRTYFSCTSAHTSTGDGTAMVTRAGLPCQDLEFVQFHPTGIYGAGCLITEGCRGEGGILINSQGERFMERYAPVAKDLASRDVVSRSMTLEIREGRGCGPEKDHVYLQLHHLPPEQLAIRLPGISETAMIFAGVDVTKEPIPVLPTVHYNMGGIPTNYKGQVLRHVNGQDQIVPGLYACGEAACASVHGANRLGANSLLDLVVFGRACALSIAESCRPGDKVPPIKPNAGEESVMNLDKLRFADGSIRTSELRLSMQKSMQSHAAVFRVGSVLQEGCEKISQLYGDLKHLKTFDRGMVWNTDLVETLELQNLMLCALQTIYGAEARKESRGAHAREDYKERIDEYDYSKPIQGQQKKLFEEHWRKHTLSYVDVQTGKVSLEYRPVIDKTLNEADCATVPPAIRSY

>Cricetulus_griseus (XP_003513683.1)

MAGVAAVSRLLRGRRLALAGVRPATFQTQTCGFHFTVGESKKASAKVSDGISTQYPVVDHEFDAVVVGAGGAGLRAAFGLSEAGFNTACLTKLFPTRSHTVAAQGGINAALGNMEEDNWRWHFYDTVKGSDWLGDQDAIHYMTEQAPASVVELENYGMPFSRTEDGKIYQRAFGGQSLKFGKGGQAHRCCCVADRTGHSLLHTLYGRSLRFDTSYFVEYFALDLLMENGECRGVIALCIEDGSIHRIRAKNTVIATGGYGRTYFSCTSAHTSTGDGTAMVTRAGLPCQDLEFVQFHPTGIYGAGCLITEGCRGEGGILINSQGERFMERYAPVAKDLASRDVVSRSMTLEIREGRGCGPEKDHVYLQLHHLPPEQLATRLPGISETAMIFAGVDVTKEPIPVLPTVHYNMGGIPTNYKGQVLKHVNGQDQVVPGLYACGEAACASVHGANRLGANSLLDLVVFGRACALSIAESCRPGDKVPPIKANAGEESVMNLDKLRFADGSIRTSELRLSMQKSMQNHAAVFRVGSVLQEGCEKISQLYGELKHLKTFDRGMVWNTDLVETLELQNLMLCALQTIYGAEARKESRGAHAREDYKVRVDEYDYSKPIQGQQKRPFEEHWRKHTLSYVDNNTGKVTLEYRPVIDKTLNETDCATVPPAIRSY

>Drosophila_melanogaster (NP_477210.1)

MSGIMRVPSILAKNAVASMQRAAAVGVQRSYHITHGRQQASAANPDKISKQYPVVDHAYDAIVVGAGGAGLRAAFGLVAEGFRTAVITKLFPTRSHTIAAQGGINAALGNMEEDDWKWHMYDTVKGSDWLGDQDAIHYMTREAPKAVIELENYGMPFSRTQDGKIYQRAFGGQSLKFGKGGQAHRCCAVADRTGHSLLHTLYGQSLSYDCNYFVEYFALDLIFEDGECRGVLALNLEDGTLHRFRAKNTVIATGGYGRAFFSCTSAHTCTGDGTAMVARQGLPSQDLEFVQFHPTGIYGAGCLITEGCRGEGGYLINGNGERFMERYAPVAKDLASRDVVSRSMTIEIMEGRGAGPEKDHVYLQLHHLPPKQLAERLPGISETAMIFAGVDVTREPIPVLPTVHYNMGGVPTNYRGQVITIDKDGKDVIVPGLYAAGEAASSSVHGANRLGANSLLDLVVFGRACAKTIAELNKPGAPAPTLKENAGEASVANLDKLRHANGQITTADLRLKMQKTMQHHAAVFRDGPILQDGVNKMKEIYKQFKDIKVVDRSLIWNSDLVETLELQNLLANAQMTIVSAEARKESRGAHAREDFKVREDEYDFSKPLDGQQKKPMDQHWRKHTLSWVCNDNGDITLDYRNVIDTTLDNEVSTVPPAIRSY

>Homo_sapiens (NP_004159.2)

MSGVRGLSRLLSARRLALAKAWPTVLQTGTRGFHFTVDGNKRASAKVSDSISAQYPVVDHEFDAVVVGAGGAGLRAAFGLSEAGFNTACVTKLFPTRSHTVAAQGGINAALGNMEEDNWRWHFYDTVKGSDWLGDQDAIHYMTEQAPAAVVELENYGMPFSRTEDGKIYQRAFGGQSLKFGKGGQAHRCCCVADRTGHSLLHTLYGRSLRYDTSYFVEYFALDLLMENGECRGVIALCIEDGSIHRIRAKNTVVATGGYGRTYFSCTSAHTSTGDGTAMITRAGLPCQDLEFVQFHPTGIYGAGCLITEGCRGEGGILINSQGERFMERYAPVAKDLASRDVVSRSMTLEIREGRGCGPEKDHVYLQLHHLPPEQLATRLPGISETAMIFAGVDVTKEPIPVLPTVHYNMGGIPTNYKGQVLRHVNGQDQIVPGLYACGEAACASVHGANRLGANSLLDLVVFGRACALSIEESCRPGDKVPPIKPNAGEESVMNLDKLRFADGSIRTSELRLSMQKSMQNHAAVFRVGSVLQEGCGKISKLYGDLKHLKTFDRGMVWNTDLVETLELQNLMLCALQTIYGAEARKESRGAHAREDYKVRIDEYDYSKPIQGQQKKPFEEHWRKHTLSYVDVGTGKVTLEYRPVIDKTLNEADCATVPPAIRSY

>Lonchura_striata_domestica (XP_021384412.1)

MAAAAAARGLARRWLRPAAASRAWPAACQAPARNLHFTAYGKKNASTKVSDSISTQYPVVDHEFDAVVVGAGGAGLRAAFGLSEAGFNTACVTKLFPTRSHTVAAQGGINAALGNMEDDNWRWHFYDTVKGSDWLGDQDAIHYMTEQAPAAVIELENYGMPFSRTEEGKIYQRAFGGQSLQFGKGGQAHRCCCVADRTGHSLLHTLYGRSLRYDTSYFVEYFALDLLMENGECRGVIALCIEDGTIHRFRAKNTVIATGGYGRTYFSCTSAHTSTGDGTAMVTRAGLPCQDLEFVQFHPTGIYGAGCLITEGCRGEGGILINSQGERFMERYAPVAKDLASRDVVSRSMTIEIREGRGCGPEKDHVYLQLHHLPPEQLATRLPGISETAMIFAGVDVTKEPIPVLPTVHYNMGGIPTNYKGQVITHVNGEDKVVPGLYACGEAASASVHGANRLGANSLLDLVVFGRACALTIANTCKPGEPVPPIKPNAGEESVANLDKLRFANGSIRTSELRLNMQKAMQNHAAVFRTGSVLQEGCEKLSQIYGDLAHLKTFDRGIVWNTDLVETLELQNLMLCALQTIYGAEARKESRGAHAREDYKLRVDEFDYSKPLQGQQRKPFEEHWRKHTLSYVDIPTGKVTLKYRPVIDKTLNEEDCQTVPPAIRSY

>Oryzias_melastigma (XP_024123839.1)

MASVRAASRLLSRRTISHVKAVPAAAVQSSRNFHFSIYGKKKNAKVSDDISTQYPVVDHEFDAVVVGAGGAGLRAAFGLSEAGFNTACVTKLFPTRSHTVAAQGGINAALGNMEDDDWRWHFYDTVKGSDWLGDQDAIHYMTEQAPAAVVELENFGMPFSRTEDGKIYQRAFGGQSLKYGKGGQAHRCCCVADRTGHSLLHTLYGRSLRYDTSYFVEYFALDLLMEDGQCKGVIALCMEDGSIHRFRAQNTVIATGGYGRTYFSCTSAHTSTGDGNAMVTRAGLPCQDLEFVQFHPTGIYGAGCLITEGCRGEGGILINSEGERFMERYAPNAKDLASRDVVSRSMTIEIREGRGVGPEKDHVYLQLHHLPPQQLATRLPGISETAMIFAGVDVTKEPIPVLPTVHYNMGGIPTNYKGQVIDYTDGKDTVVPGLYACGEAACASVHGANRLGANSLLDLVVFGRACALTIANEHKPGEKLSPLKPSAGEESVANLDKLRFANGSLRTSEIRLNMQKTMQNHAAVFRTGSVLKEGCDKMDDIYQTMEQIKTFDRGIVWNTDLVESLELQNLMLNAVQTINSAEQRKESRGAHAREDFRDRVDEFDYSKPLQGQEKKPFDQHWRKHTLSYVDPKTGKVTLKYRPVIDTSLDEQDCAHVPPAIRSY

>Gekko_japonicus (XP_015276221.1)

MAAAAVRGWRRPLRAFRWPGPAACQAFSRQFHFTVYGKKSASAKVSDSISTQYPVVDHEFDAVVVGAGGAGLRAAFGLSEAGFNTACVTKLFPTRSHTVAAQGGINAALGNMEEDNWRWHFYDTVKGSDWLGDQDAIHYMTEQAPAAVIELEKYLKFGKGGQAHRCCCVADRTGHSLLHTLYGRSLRYDTSYFVEYFALDLLMENGACHGVVALCIEDGTIHRLRAKNTVLATGGYGRTYFSCTSAHTTTGDGTAMVTRAGLPCQDLEFVQFHPTGIYGAGCLITEGCRGEGGILINSEGERFMERYAPVAKDLASRDVVSRSMTIEIREGRGCGPEKDHVYLQLHHLPPQQLAMRLPGISETAMIFAGVDVTREPIPVLPTVHYNMGGIPTNYKGQVITHVNGQDQVVPGLYACGEAASASVHGANRLGANSLLDLVVFGRACALSIAESCKPGEPVPSVKPNAGEESVANLDKLRFANGNLRTSELRLNMQKTMQTHAAVFRTGSVLQEGCEKLSSIYKDLDNLKTFDRGVVWNTDLVETLELQNLMLCALQTIYGAEARKESRGAHAREDYKVRIDEYDYSKPTEGQQKKPFDQHWRKHTLSYVDVGTGKVTLEYRPVIDNTLNEEDCAMVPPAIRSY

>Neophocaena_asiaeorientalis_asiaeorientalis (XP_024587721.1)

MSGIGVLSRMLRARRLALTWAQRPEALHAGARSFHFTVDSSKRSSAKVSDAISTQYPVVDHEFDAVVVGAGGAGLRAAFGLSEAGFNTACVTKLFPTRSHTVAAQGGINAALGNMEEDNWRWHFYDTVKGSDWLGDQDAIHYMTEQAPASVVELENYGMPFSRTEDGKIYQRAFGGQSLKFGKGGQAHRCCCVADRTGHSLLHTLYGRSLRYDTSYFVEYFALDLLMENGECRGVIALCIEDGSIHRIRAKNTVVATGGYGRTYFSCTSAHTSTGDGTAMITRAGLPCQDLEFVQFHPTGIYGAGCLITEGCRGEGGILVNSQGERFMERYAPVAKDLASRDVVSRSMTLEIREGRGCGPEKDHVYLQLHHLPPEQLAMRLPGISETAMIFAGVDVTKEPIPVLPTVHYNMGGIPTNYKGQVLKHVGGQDQVVPGLYACGEAACASVHGANRLGANSLLDLVVFGRACALGIAESCKPGDKVPSIKPNAGEESVMNLDKLRFANGSIRTSELRLNMQKSMQSHAAVFRVGSVLQEGCEKISKLYGDLKHLKTFDRGMVWNTDLVETLELQNLMLCALQTIYGAEARKESRGAHAREDFKERVDEYDYSKPIQGQHKRPFEGHWRKHTLSYVDIRTGKVSLEYRPVIDRTLNEADCATVPPAIRSY

**OCEANIA**

>Crocodylus_porosus (YP_637149.1)

MLKIIVPTMMLIPSTCLTATKNTWLSPTAYSAVIIILGMLVLNPGDTLMNTTGLLLGSDQISTPLLMLSCWLLPLMFMASQSSMSHNPAQQKRLFITALALLQLALMLVFMALDLMLFYTTFEATLIPTLMVIARWGSQTERLGAGLYFLLYTITSSMPLLIALLWVYNMKGTASITLLQLLPPMTLTFWTNTLLWTSLMLAFLVKIPIYGLHLWLPKAHVEAPIAGSMVLAAILLKLGGYGLLRITNLLTEQTTSSYILPLAVALWGALMTGMVCLRQTDLKSLIAYSSVSHMGLMTSSILTHNQLAPSGSMIMMVAHGLTSSMLFCLANINYERTHSRTLLLTQGVQLTTPAMTSWWLLACLTNMALPPTINFIGELTLMVSLFDWADITIFLTGLSAFITSIYTLHMFSSTQQGTLPTHIITMSPTQTREHLLMTLHSAPSIALIFMPQLMYYQ

>Drosophila_melanogaster (YP_009047274.1)

MLKIIFFLLFLIPFCFINNMYWMVQIMMFFISFIFLLMNNFMNYWSEISYFLGCDMLSYGLILLSLWICSLMLLASEMINKHNNYKNLFLLNIIILLLLLILTFSSMSLFMFYLFFESSLIPTLFLILGWGYQPERLQAGLYLLFYTLLVSLPMLIGIFYLMNKIGSMNFYLMNNFMFNYDLLYFCLLCAFLVKMPMFLVHLWLPKAHVEAPVSGSMILAGIMLKLGGYGMLRVISFLQLMNLKYSFVWISISLVGGVLVSLVCLRQTDLKALIAYSSVAHMGIVLSGLLTMTYWGLCGSYTLMIAHGLCSSGLFCLANVSYERLGSRSMLINKGLLNFMPSMTLWWFLLSSANMAAPPTLNLLGEIYLLNSIVSWSWISMILLSFLSFFSAAYTLYLYSFSQHGKLFSGVYSFSSGKIREYLLMLLHWLPLNLLILKSESFMLWL

>Homo_sapiens (YP_003024035.1)

MLKLIVPTIMLLPLTWLSKKHMIWINTTTHSLIISIIPLLFFNQINNNLFSCSPTFSSDPLTTPLLMLTTWLLPLTIMASQRHLSSEPLSRKKLYLSMLISLQISLIMTFTATELIMFYIFFETTLIPTLAIITRWGNQPERLNAGTYFLFYTLVGSLPLLIALIYTHNTLGSLNILLLTLTAQELSNSWANNLMWLAYTMAFMVKMPLYGLHLWLPKAHVEAPIAGSMVLAAVLLKLGGYGMMRLTLILNPLTKHMAYPFLVLSLWGMIMTSSICLRQTDLKSLIAYSSISHMALVVTAILIQTPWSFTGAVILMIAHGLTSSLLFCLANSNYERTHSRIMILSQGLQTLLPLMAFWWLLASLANLALPPTINLLGELSVLVTTFSWSNITLLLTGLNMLVTALYSLYMFTTTQWGSLTHHINNMKPSFTRENTLMFMHLSPILLLSLNPDIITGFSS

>Lechriodus_melanopyga (YP_007316801.1)

MLTAVFAFLTMILTTYLAPTNLLWALVTAQSFIIAVLSFLWFTSSEILPTLNQYLLVDEISSPLLILTFWLTPLTLLASQSKISKEPASRQRSYIFSILFLQITTALAFLANNLMLFFIFFESTMIPTLIVITRWGTQKERIMAGIYMLFYTLAGSLLLLTAILYFHESFGSLTISMLKKIQEEQMINFSSTIWWLACLVAFLVKMPLYGLHLWLPKAHVEAPIAGSMILAGTLLKLGGYGLLRMSTLINESMYALATPLIIFSMCGVVASALLCSRQTDLKSLIAFSSVSHMGLVVAASMIKSPWSVSGAMILMISHGLISSALFCLANTAYERTSTRTMVVLQGSQVMLPLAAAWWLLSVMLNMALPPSTNFMGELLILQSIFKWAPYSIILAGLGIIFTTSYSLYLFWSSQREHLPTHLKSMPPTVTREHILLALHILPIIFLIIKPELIF

>Maccullochella_macquariensis (YP_009175624.1)

MLKILIPTLMLIPTAYTTKAKWLWPTTLFYSLTIALTSLPLLKNLSETGWSSLGLYMATDNLSTPLLILTCWLLPLMILASQKHTTFEPINRQRTYIALLTSLQLFLILAFSATELIMFYIMFEATLIPTLILITRWGNQMERLNAGTYFLFYTLAGSLPLLVALMLIQKNTGTLSLLTLQYSNPVPMLTYADKLWWAGCLLAFLVKMPLYGVHLWLPKAHVEAPIAGSMILAAVLLKLGGYGMMRMMVMLEPLTKELSYPFLIFALWGVIMTGSICLRQTDLKSLIAYSSVSHMGLVAGGILVQTSWGFTGALILMIAHGLTSSALFCLANTNYERTHSRTLMLTRGLQMALPLMTTWWFITSLANLALPPLPNLIGELMIIISLFNWSWWTIALTGTGTLITAGYSLYMFLTTQRGQLPTHILTMDPSHTREHLLITLHLLPLILLTFKPELISGWTS

>Phascolarctos_cinereus (YP_637019.1)

MLKILMPTFMLIPLTWLSKNLWLWTNLTSYSLLIGTFSITLLHQDSDLGTNHNNLFYTDSLSSPLLVLSCWLLPLMMMASQNHLNKESMNRKKAYLTTLIILQLSLITALSASELMMFYIMFETTLIPTLIIITRWGNQNERLNAGLYLLFYTLTGSIPLLIALLFLYNKLGSLHILAMTIMSTTLKPSYSNSILWYACMTAFMVKMPLYGLHLWLPKAHVEAPIAGSMVLAAILLKLGGYGIMRMTVFTQPLTTNLYYPFIILSLWGMVMTSFICLRQTDLKSLIAYSSISHMALVIIATLMQSPLSFMGATALMIAHGLTSSMLFGLANTNYERINSRTMILARGLQTILPLTCTWWILATLANLALPPTINFLCELLVITSSFSWSNFSIILLGINTIITALYSLHMLTTTQRGKPTYHTQTIKPTSTREHTLMILHLIPLLTISLSPKFILGLTY

>Sus_scrofa (NP_008643.1)

MLKIIIPTTMLLPMTWMSKHNMIWINATVHSLLISLISLSLLNQLGENSLNFSLTFFSDSLSAPLLVLTTWLLPLMLMASQSHLSKETTTRKKLYITMLILLQLFLIMTFTATELILFYILFEATLVPTLIIITRWGNQTERLNAGLYFLFYTLAGSLPLLVALVYIQNTTGSLNFLIIHYWSHPLSNSWSNIFMWLACIMAFMVKMPLYGLHLWLPKAHVEAPIAGSMVLAAVLLKLGGYGMMRITTILNPLTNYMAYPFLMLSMWGMIMTSSICLRQTDLKSLIAYSSVSHMALVIVAIMIQTPWSFMGATALMIAHGLTSSMLFCLANTNYERVHSRTMILARGLQTLLPLMATWWLMASLTNLALPPSINLIGELFIITASFSWSNITIILMGMNMMITALYSLYMLITTQRGKYTHHINNIKASFTRENALMALHILPLLLLTLNPKMILGPLY

>Tregellasia_capito (YP_009144002.1)

MLKILIPTAMLLPLALCSPRKHLWTNTTAYSLLIAAASLQWLTPTYYPNKNLSNWAAIDQISSPLLVLSCWLLPLMIMASQNHLEQEPTIRKRIFITTLLLAQPFILTAFSASELMLFYIAFEATLIPTLILITRWGSQPERLTAGIYLLFYTLASSLPLLIAILHLQNQIGSLSFMMLKLTHPTMTSSWTSLVTGLALLLAFMVKAPLYGLHLWLPKAHVEAPIAGSMLLAALLLKLGGYGIMRVTMLVNPSLNNLHYPFITLALWGALMTSTICLRQIDLKSLIAYSSVSHMGLVVAATMIQTQWAFSGAMMLMISHGLTSSMLFCLANTNYERTHSRILLLTRGLQPLLPLMATWWLLANLTNMALPPTTNLMAELTIAIALFNWSSLTIILTGGTILLTTSYTLYMLTMTQRGTIPPHITSIQNSSTREHLLMALHVIPMALLILKPELIAGIPM

**AFRICA**

>Acinonyx_jubatus (NP_941390.1)

MTNIRKSHPLIKIVNHSFIDLPTPPNISAWWNFGSLLGVCLVLQILTGLFLAMHYTSDTMTAFSSVTHICRDVNYGWIIRYMHANGASMFFICLYMHVGRGMYYGSYTFSETWNIGIMLLLTVMATAFMGYVLPWGQMSFWGATVITNLLSAIPYIGTNLVEWIWGGFSVDKATLTRFFAFHFILPFIISALAAVHLLFLHETGSNNPSGITSDSDKIPFHPYYMIKDILGLLMLILMLTLLVLFSPDLLGDPDNYIPANPLNTPPHIKPEWYFLFAYAILRSIPNKLGGVLALMFSILILAIIPIFHTSKQRGMMFRPLSQCLFWLLVADLLTLTWIGGQPVEHPFITIGQLASILYFSTLLVLMPISGIIENRLLKW

>Drosophila_melanogaster (YP_009047277.1)

MNKPLRNSHPLFKIANNALVDLPAPINISSWWNFGSLLGLCLIIQILTGLFLAMHYTADINLAFYSVNHICRDVNYGWLLRTLHANGASFFFICIYLHVGRGIYYGSYKFTPTWLIGVIILFLVMGTAFMGYVLPWGQMSFWGATVITNLLSAIPYLGMDLVQWLWGGFAVDNATLTRFFTFHFILPFIVLAMTMIHLLFLHQTGSNNPIGLNSNIDKIPFHPYFTFKDIVGFIVMIFILISLVLISPNLLGDPDNFIPANPLVTPAHIQPEWYFLFAYAILRSIPNKLGGVIALVLSIAILMILPFYNLSKFRGIQFYPINQVMFWSMLVTVILLTWIGARPVEEPYVLIGQILTVVYFLYYLVNPLITKWWDNLLN

>Homo_sapiens (YP_003024038.1)

MTPMRKTNPLMKLINHSFIDLPTPSNISAWWNFGSLLGACLILQITTGLFLAMHYSPDASTAFSSIAHITRDVNYGWIIRYLHANGASMFFICLFLHIGRGLYYGSFLYSETWNIGIILLLATMATAFMGYVLPWGQMSFWGATVITNLLSAIPYIGTDLVQWIWGGYSVDSPTLTRFFTFHFILPFIIAALATLHLLFLHETGSNNPLGITSHSDKITFHPYYTIKDALGLLLFLLSLMTLTLFSPDLLGDPDNYTLANPLNTPPHIKPEWYFLFAYTILRSVPNKLGGVLALLLSILILAMIPILHMSKQQSMMFRPLSQSLYWLLAADLLILTWIGGQPVSYPFTIIGQVASVLYFTTILILMPTISLIENKMLKWA

>Erpetoichthys_calabaricus (NP_943616.1)

MAIMRKTHPLAKIINSAFIDLPAPSNISSWWNMGSLLGLCLIVQIITGLFLAMHYISDINLAFSSVAHICRDVNYGWLIRNIHANSASLFFICIYLHIARGLYYGSYLYMETWNVGVILLLLTMMTAFVGYVLPWGQMSFWGATVITNLLSAVPYIGDTLVQWIWGGFSVDKPTLTRFFAFHFILPFAIAGASLVHILFLHETGSNNPLGINSNADKIPFHPYYTYKDLLGFIILLLIILMLALLSPNLLNDPENFTPANPLVTPPHIKPEWYFLFAYAILRSIPNKLGGVLALLFSIIVLMFVPFLHTAKIRTSTFRPLFKITLWILAADVMILTWIGGQPVEDPYIMIGQVASVLYFTIFLVFMPVSGWIENKMMNRN

>Fukomys_damarensis (YP_009163380.1)

MTNIRKSHPLIKIINHSFIDLPTPSSISYWWNFGSLLGACLILQIITGLFLSMHYTADTATAFSSVAHICRDVNYGWLIRYLHANGASMFFICLYLHVGRGMYYGSYMFMETWNIGIILLLSVMATAFMGYVLPWGQMSFWGATVITNLFSAIPYIGPTLVEWIWGGFAVDKATLTRFFAFHFILPFIITALTMVHLLFLHETGSNNPSGINSDSDKIPFHPYYSFKDFMGLQIMLLILLTLTLFHPDLLGDPDNYTPANPMSTPPHIKPEWYFLFAYAILRSIPNKLGGVLALVMSILILVALPLLHTSKQRSMMFRPISQCLFWTFISTLLTLTWIGSQPVEYPYIIIGQLASILYFLIILVLMPLAGLVENKMMKW

>Numida_meleagris (YP_009357218.1)

MAPNIRKSHPLLKMINNSLIDLPTPSNISAWWNFGSLLAVCLMTQIITGLLLAMHYTADTSLAFSSVAHTCRNVQYGWLIRNLHANGASFFFICIYLHIGRGLYYGSYLYKETWNTGVILLLTLMATAFVGYVLPWGQMSFWGATVITNLFSAIPYIGQTLVEWAWGGFSVDNPTLTRFFALHFLLPFVIAGITIIHLTFLHESGSNNPLGISSNSDKIPFHPYYSIKDILGLTLMLTPLLTLALFSPNLLGDPENFTPANPLVTPPHIKPEWYFLFAYAILRSIPNKLGGVLALAASVLILLLIPFLHKSKQRTMTFRPFSQLLFWLLVANLLILTWVGSQPVEHPFIIIGQLASLSYFTTLLILFPMIGTLENKMLNH

>Xenopus_laevis (NP_008146.1)

MAPNIRKSHPLIKIINNSFIDLPTPSNISSLWNFGSLLGVCLIAQIITGLFLAMHYTADTSMAFSSVAHICFDVNYGLLIRNLHANGLSFFFICIYLHIGRGLYYGSFLYKETWNIGVILLFLVMATAFVGYVLPWGQMSFWGATVITNLLSAKPYIGNVLVQWSLGGFSVDNATLTRFFAFHFLLPFIIAGASILHLLFLHETGSTNPTGLNSDPDKVPFHPYFSYKDLLGFLIMLTALTLLAMFSPNLLGDPDNFTPANPLITPPHIKPEWYFLFAYAILRSMNKLGGVLALVLSILILALMPLLHTSKQRSLMFRPFTQIMFWALVADTLILTWIGGQPVEDPYTMIGQLASVIYFSIFIIMFPLMGWVENKLLNW

>Giraffa_giraffa (ABQ15230.1)

MINIRKSHPLMKIVNNALIDLPAPSNISSWWNFGSLLGICLILQILTGLFLAMHYTPDTTTAFSSVTHICRDVNYGWIIRYMHANGASMFFICLFMHMGRGLYYGSYTFLETWNVGMILLFTVMATAFMGYVLPWGQMSFWGATVITNLLSAIPYIGTNLVEWIWGGFSVDKATLTRFFAFHFILPFIIAALTMVHLLFLHETGSNNPMGITSDMDKIPFHPYYTIKDILGALLLILTLMLLVLFTPDLLGDPDNYTPANPLNTPPHIKPEWYFLFAYAILRSIPNKLGGVLALIFSILILALMPLLHTSKQRSMTFRPLSQCLFWILVADLLTLTWIGGQPVEHPFIIIGQLASIMYFLIILVLMPVTSAIENNLLKW
