# Supplementary material for: Using active learning methodologies to teach sequence analysis and molecular phylogeny
Source: Biochem Mol Biol Educ. 2024 Oct 14;53(1):21–32. doi: 10.1002/bmb.21861 (PMC11752413; doi:10.1002/bmb.21861)
Supplement: Supplementary file 7 — Data S3. Doc.3 Supplementary material. DNA sequences provided in this work. [file BMB-53-21-s005.docx]

**AMERICA**

>Ambystoma_tigrinum (NC_006887.1)

ATGAACCTAAGCTTTTTTGACCAATTCATAAGCCCAATTATGTTAGGTATTCCATTAATTTTACTAGCAATAACTATTCCATGATTATTATATGCTTCACCAACAGATCGATGATTAAATAACCGCCTTACCACCCTACAAGCATGATTTTTAGCCTCCTTTACAAAACAACTAATATTACCTCTGAACATTAAAGGACATAAATGAGCCCTACCATTGACTTCACTAATAATCTTTTTAATTACAATAAATTTATTAGGGTTGTTACCTTATACTTTTACCCCGACAACTCAACTCTCATTAAATTTAGGATTAGCTGTTCCATTCTGACTAGCTACAGTACTAATTGGATTACGAAACCAACCAACTGCAGCACTAGGACACTTACTTCCTGAGGGTACTCCAACACTACTAATCCCAATTTTAATTATTATTGAAACAATTAGCCTATTTATCCGCCCATTAGCTTTAGGGGTTCGTCTTACTGCTAACCTTACAGCTGGCCATCTTCTTATTCAACTTATTTCTACAGCAGTATTTGTTTTAATACCAATAATACCAACAACAGCTATTATTACTGCTATTGTATTATTTCTTTTAACTCTTTTAGAAATTGCCGTAGCAATAATTCAAGCTTATGTCTTTGTCCTACTATTAAGCCTCTATCTTCAAGAAAACACATAA

>Caiman_crocodilus (NC_002744.2)

ATGAACACTAACCTATTTGACCAATTCATAATCCCCAACCTCATAGGCACCCCCCTACTCATACCTGCCCTATTAATCATTCCCCTCCTCCTCCTAAACCCAAAAAACCAATGACTATCAAACCCAGCAACAACAATAAAATCTTGATACATTACCCAAATCACCAAACAAATCATAACCCCAATCAATAAACCCGGACACATACACTCCGTTACCCTCATCTCCTTACTAATCCTCCTCTCTTTCACAAACCTATTAGGACTACTCCCATATACATTTACCCCAACAACACAACTATCTATAAACATAGCCCTTGCCCTCCCCCTATGGGGAATAACCGTACTAATTGGACTACGAACCCAACCAACAACCTCCCTTGCTCACCTCCTCCCAGAAGGAACCCCAACCCCATTAATCCCCATCCTCATCCTAATTGAAACAATCAGCCTACTAATCCGACCTGTTGCCCTAGCCGTACGACTAACAGCAAATCTAACCGCAGGCCACTTACTTATTCAATTACTCTCTATAGCCACAATAAACCTATGGTCTATTATACCCCCTCTCAGCCTACTAACACTAACAACCCTAACCCTCCTCCTTCTCCTAGAGTTCGCCGTAGCCATAATCCAAGCATACGTTTTTGTCCTATTACTCTCCCTATACCTACAAGAAAACACATAA

>Drosophila_melanogaster (NC_024511.2)

ATGATAACAAATTTATTTTCTGTATTCGACCCCTCAGCTATTTTTAATTTTTCACTTAATTGATTAAGAACATTTTTAGGACTTTTAATAATTCCGTCAATTTATTGATTAATACCTTCTCGTTACAATATTATATGAAATTCAATTTTATTAACTCTTCATAAAGAATTTAAAACTTTATTAGGCCCATCAGGTCATAATGGATCTACTTTTATTTTTATTTCTTTATTTTCATTAATTTTATTTAATAATTTCATAGGATTATTTCCATATATTTTTACAAGAACAAGACATTTAACTTTAACTTTATCTTTAGCTTTACCTTTATGATTATGTTTTATATTATATGGATGAATTAATCATACACAACATATATTTGCTCATTTAGTTCCTCAAGGAACACCCGCTATTCTTATACCTTTTATAGTATGTATTGAAACTATTAGAAATATTATTCGACCTGGAACATTAGCTGTTCGATTAACTGCTAATATAATTGCTGGACATTTATTATTAACTCTTTTAGGAAATACAGGACCTTCTATATCTTATATTTTAGTAACATTTTTATTAATAGCTCAAATTGCTTTATTAGTATTAGAATCAGCTGTAGCTATAATTCAATCTTATGTGTTTGCTGTATTAAGAACTTTATATTCTAGAGAAGTAAATTAA

>Himatione_sanguinea (NC_025602.1)

ATGAACCTAAGCTTCTTCGACCAATTCTCAAGCCCATCCTTCCTAGGAATCCCACTCATCCTCATCTCAATGACATTTCCAGCCCTCCTAATCCCCTCACTAGACAACCGATGAATCACTAACCGACTCTCAACCCTCCAACTATGATTTGTCAATCTAGTCACAAAACAACTAATAATGCCCTTAGACAAAAAAGGTCATAAATGAGCCCTAATCTTAACATCCCTAATAATCTTCCTGCTACTAATCAACCTCCTAGGCCTACTACCATATACATTCACCCCAACTACCCAATTATCTATAAACTTAGCCCTAGCTTTCCCCCTATGACTTGCTACCCTACTAACAGGCCTGCGAAACCAACCCTCCATCTCGCTAGGACACCTCCTCCCAGAAGGCACTCCAACCCCACTAATCCCTGCCCTAATTTTAATCGAAACGACAAGCCTACTAATCCGACCCCTAGCCCTAGGCGTACGCCTGACAGCTAACCTAACAGCAGGCCACCTGCTTATTCAACTCATCTCCACAGCCACAACAGCCCTACTCCCTACAATACCAGCAGTCTCACTTCTAACCCTATTAGTTCTATTCTTACTGACTATCCTAGAAGTAGCAGTAGCAATGATTCAAGCTTACGTCTTCGTACTCCTACTCAGCCTCTACCTACAAGAGAACATCTAA

>Marmota_flaviventris (NC_042243.1)

ATGAACGAAAATCTATTCGCCTCTTTCATTACCCCTACATTAATAGGCTTTCCTATTGTCCTTTTTATTATTATATTTCCCAATTTACTCTTCCCTTCCCCTACTCGATTAGTAAATAACCGTCTAGTATCATTTCAACAATGACTAATCCAACTTGTATTAAAACAAATAATGACAATTCATAACCCAAAAGGACGTACCTGATCCCTAATACTAATCTCATTAATTATATTTATTGGCTCTACTAATCTTCTAGGATTATTACCCCACTCTTTTACACCAACTACTCAGCTATCAATAAATTTAGGAATAGCTATTCCTCTATGAGCAGGAGCAGTAATTACCGGATTTCGTTATAAAACCAAAGCATCATTAGCCCACTTTCTCCCACAAGGAACTCCAATTTTACTTATTCCTATACTTATTATTATCGAAACAATCAGCCTTTTCATTCAACCCATAGCATTAGCCGTGCGACTAACAGCTAATATCACAGCCGGCCATCTTCTCATGCATTTAATTGGAGGGGCAACTCTTGTATTAACATCTATTAGCCCTCCTACAGCTATTCTAACTTTTACTATTCTTGTATTACTAACAATGCTTGAATTTGCAGTTGCATTAATTCAAGCTTACGTTTTCACTCTCCTAGTAAGCTTATATTTACATGATAATACTTAA

>Homo_sapiens (NC_012920.1)

ATGAACGAAAATCTGTTCGCTTCATTCATTGCCCCCACAATCCTAGGCCTACCCGCCGCAGTACTGATCATTCTATTTCCCCCTCTATTGATCCCCACCTCCAAATATCTCATCAACAACCGACTAATCACCACCCAACAATGACTAATCAAACTAACCTCAAAACAAATGATAACCATACACAACACTAAAGGACGAACCTGATCTCTTATACTAGTATCCTTAATCATTTTTATTGCCACAACTAACCTCCTCGGACTCCTGCCTCACTCATTTACACCAACCACCCAACTATCTATAAACCTAGCCATGGCCATCCCCTTATGAGCGGGCACAGTGATTATAGGCTTTCGCTCTAAGATTAAAAATGCCCTAGCCCACTTCTTACCACAAGGCACACCTACACCCCTTATCCCCATACTAGTTATTATCGAAACCATCAGCCTACTCATTCAACCAATAGCCCTGGCCGTACGCCTAACCGCTAACATTACTGCAGGCCACCTACTCATGCACCTAATTGGAAGCGCCACCCTAGCAATATCAACCATTAACCTTCCCTCTACACTTATCATCTTCACAATTCTAATTCTACTGACTATCCTAGAAATCGCTGTCGCCTTAATCCAAGCCTACGTTTTCACACTTCTAGTAAGCCTCTACCTGCACGACAACACATAA

>Pterois_volitans (NC_025290.1)

ATGACACTAAGCTTCTTTGATCAATTTATAAGCCCAACCTATCTAGGGATCCCATTAATAGCCCTTGCTCTCACTCTTCCTTGAATTTTGTTTCCCACTCCGACGGCCCGATGATTAAACAACCGCCCTATGACTTTGCAAGGATGGTTTATTAACCGTTTTACACAACAACTACTACTTCCCCTCAACGTAGGGGGCCACAAGTGAGCTGCCCTCCTGGCTTCATTAATGATTTTCTTAATTACACTAAATATACTGGGACTTCTACCATACACTTTCACCCCAACAACGCAATTGTCACTTAATTTAGGGCTGGCAGTACCACTTTGATTAGCAACCGTTATTATTGGCATGCGAAACCAACCCACCCATGCTCTCGGACACCTTCTGCCAGAAGGCACACCAGGCCCACTGATCCCCATCCTAATTATTATCGAAACAATTAGCTTATTTATTCGACCACTAGCGCTGGGAGTACGACTAACAGCCAATCTCACAGCAGGACACCTACTCATTCAACTAATCGCCACCGCCGCCTTCGTACTACTACCTCTTATGCCCTCCGTAGCCATCCTCACAACGACAGTCCTGGTCCTCCTGACCTTACTAGAGATTGCCGTGGCAATAATCCAAGCATATGTATTCGTACTATTATTAACCCTCTACCTACAAGAAAACGTCTAA

>Ursus_americanus (NC_003426.1)

ATGAACGAAAGTTTGTTCACCTCTTTTATTACCCCAACAATAATGGGAATTCCTATTGTAGTATTAATCATTATGTTTCCGAGCATTCTATTTCCTTCTCCTAGTCGACTAGTCAATAACCGCTTAGTATCTATTCAACAGTGACTAGTCCGACTAACATCAAAACAAATACTTTCCATTCATAATCATAAAGGACAAACCTGAGCACTAATGTTGATGTCACTAATCCTATTTATTGGCTCGACCAATCTTCTGGGTCTGTTGCCACACTCATTCACACCCACCACACAACTGTCAATGAATCTGGGAATAGCTATTCCCTTGTGGACAGGCACAGTTGCTATCGGACTTCGATACAAGACCAAGGCATCCTTGGCTCACTTTCTACCTCAAGGAACACCTTTTCCCCTAATCCCTATACTTGTAATTATCGAAACGATCAGCCTATTCATCCAACCTATAGCCTTAGCCGTTCGACTAACCGCTAACATTACTGCAGGTCACCTGCTCATCCATTTGATTGGGGGAGCTACTCTGGCCCTAACAAGTATTAGCACCATTACAGCCCTTATCACCTTTACCATTCTAGTGCTACTCACTATTCTTGAGTTCGCTGTAGCCCTCATTCAGGCCTACGTCTTCACTCTACTAGTAAGCCTATACTTACACGATAACACCTAA

**ASIA**

>Ailuropoda_melanoleuca (XM_002926640.4)

GCACTGTCCCATCGTCCTGGCCGGGCGCGGCTAGATGCGCAGGCGCAGTCTGACGCGGGGGCTGGCGGGACAGCCAGCAGGAGGACAGACATGTCGGGGGTCCGGGCGGTGTCTCGGCTGTTGGGCGCCCGGCGCCTGGCACTGACCAGGGCGCAGTGGCCAACGACAAGGCAGACAGGCTCCCGCGGTTTTCACTTCACTGTTGATGGGAATAAGAGGTCTTCTGCGAAAGTTTCCGATTCAATTTCTACGCAGTACCCAGTAGTGGACCATGAGTTCGATGCGGTGGTGGTAGGTGCTGGAGGGGCAGGCCTGCGAGCTGCGTTTGGCCTTTCCGAGGCTGGGTTTAACACGGCCTGCGTCACAAAGCTCTTTCCTACCAGATCACACACCGTTGCAGCCCAGGGAGGAATCAATGCTGCTCTGGGGAACATGGAGGAAGACAACTGGAGGTGGCACTTCTACGACACCGTGAAGGGCTCCGACTGGCTGGGGGACCAGGACGCCATCCACTACATGACAGAGCAGGCACCTGCTTCCGTGGTAGAGCTGGAGAATTATGGCATGCCATTTAGCAGAACTGAAGATGGGAGGATTTACCAGCGTGCTTTTGGCGGACAGAGCCTCAAGTTTGGGAAAGGCGGGCAGGCCCACCGGTGCTGCTGTGTGGCTGACCGCACCGGCCACTCACTGCTGCACACGTTGTATGGAAGGTCTCTGCGATATGATACCAGCTATTTTGTAGAGTATTTTGCTTTGGATCTCCTGATGGAAAATGGGGAGTGTCGTGGTGTTATCGCACTGTGCATAGAGGATGGGTCCATCCATCGCATAAGAGCCAAGAACACTGTTGTAGCTACCGGAGGCTACGGGCGTACCTACTTCAGCTGCACCTCTGCCCACACCAGCACCGGTGATGGCACCGCCATGGTCACCAGGGCAGGCCTTCCCTGCCAGGACTTGGAGTTTGTTCAGTTCCACCCCACAGGTATATATGGTGCTGGTTGTCTCATTACGGAAGGGTGCCGTGGAGAAGGAGGCATTCTCATTAACAGCCAAGGTGAAAGGTTCATGGAGCGATATGCCCCAGTCGCAAAGGACCTGGCGTCCAGAGACGTTGTATCTCGGTCCATGACTCTGGAAATCCGTGAAGGAAGAGGCTGTGGCCCTGAGAAAGATCACGTCTACCTGCAGTTGCACCACCTACCCCCCGAGCAGCTGGCCATACGCCTGCCTGGCATTTCAGAGACAGCCATGATCTTCGCAGGCGTGGATGTCACCAAGGAGCCGATCCCTGTCCTTCCCACTGTGCATTATAACATGGGTGGTATTCCCACCAACTACAAGGGCCAGGTTCTGAGGCATGTGAATGGCCAGGATCAGATCGTGCCCGGCCTGTATGCCTGTGGGGAGGCCGCCTGTGCCTCAGTGCACGGTGCCAACCGTCTCGGGGCAAACTCGCTCTTGGACCTGGTCGTCTTCGGCCGGGCGTGTGCCCTGAGTATTGCAGAGTCCTGCAGACCCGGAGATAAAGTTCCCCCAATTAAACCAAATGCTGGGGAAGAGTCTGTTATGAATCTTGACAAATTGAGATTTGCTGATGGAAGCATAAGAACATCAGAACTACGACTCAGCATGCAGAAGTCGATGCAAAGTCATGCCGCAGTGTTTCGTGTGGGAAGTGTGTTACAGGAAGGATGTGAGAAAATCAGCCAGCTCTATGGAGACCTAAAACATCTGAAGACATTTGACCGAGGAATGGTCTGGAACACCGACCTGGTGGAGACCCTGGAACTGCAGAACCTGATGCTCTGTGCGCTTCAGACCATTTACGGAGCAGAGGCCCGGAAGGAGTCTCGTGGTGCTCATGCCAGGGAAGACTACAAGGAGCGGATTGACGAGTATGATTACTCCAAGCCCATTCAGGGGCAGCAGAAAAAGCTGTTTGAGGAGCACTGGCGGAAGCACACCCTTTCCTATGTTGATGTCCAGACTGGAAAGGTTTCATTGGAATACAGACCAGTGATCGACAAAACTTTAAATGAGGCTGACTGTGCCACAGTCCCTCCAGCCATTCGCTCCTACTGATGAGACCAGAGTTCACCAGCTTTTGTAATTATGTATAATAGCTCATGCGTGTGTTCATATCATAATTATCTTTTTAAAATCCTGGACTCAAGTGAACAAGGAGTGCCATTTGAATAGAGATCATCTACCCGGTGGCCAGAAGCTTGCCAGTAATGAACAGCCAGGAAGTGTGAATGAAGCTTACCTTTATCCTTTGCTTCATTCTTGTGAAATAGTCAAACTGGGCATGATTCTTAAATAAAGCATAAATTACAAACTTCTATTTCCCAGTCCATTTAAGAGCTTATACTGATTGATTTGAAGCATGTTTGTTGACCTAAAATCCAGAAATCCAGAAATTTTGTTGTATAAAGTAAAAATGAATTTTGGTGTTGACAGCACAAGAAGAAAACAGACATTTCCTCTTTGTTCTTAGTAAGCAAATGGTTTAATATGTGAACATGTGTCCTAAAGTATTTAAATAGCCAGATAGTAAAGGAAACAGATGAGGTGTAAATGGGTTTCTTCCTTCCCCGCTTGCTTTCAGGTGCTGCTTGGGGTCCGTTTCCTGCTTTGTGCTTGTCTCAGGGCTGGCTTCTGTGGGATCCAGTCAACAGTTAGGGTCATTGATTTTACTGTTTCTCAATTTTAGGGATTATCTGGTAAATATGTAAAACATTTACATGTTTTCAGAGTCCACTGTATTCAGCGCTAACTTTGATTGTCCCTGCATCCTCTCCCCTCTATCCCCCAAAGCAACCACTTTAGTTACCTTCCTAGTGAGTGTTTTAAGAGAAAAAGAAAACAAATCTGTGTTTGCATTATTGCACACACCTTGTCTGCATACCTGAAGGTCATTCCTTATCATCAGTGAGCCTTCCTTCTTTTGGCTGCATGAATGTACCTTACCCCCAGTGGTGGACGTTTGCGTTCTTTCCAGTATTACAAATGCCACAATGAG

>Cricetulus_griseus (XM_003513635.5)

TTAAACCCGACGCCGGCGTAACGTGGTTGCGCAAGCGCGGTTGTCGCTGAGGCTTGTGGGACTGCAGAAACATGGCTGGCGTTGCAGCGGTTTCGCGGCTTCTTCGCGGGCGCCGCTTGGCGCTAGCTGGGGTGCGGCCAGCAACATTCCAAACACAGACCTGTGGCTTTCATTTCACTGTTGGTGAAAGCAAGAAGGCATCTGCCAAAGTTTCAGATGGGATTTCTACTCAGTACCCAGTTGTGGATCATGAATTTGATGCTGTGGTGGTAGGCGCTGGAGGGGCAGGCTTGAGAGCTGCATTTGGACTTTCTGAGGCAGGGTTTAATACTGCATGCCTTACAAAGCTCTTTCCGACCCGATCACATACTGTTGCAGCACAGGGAGGCATCAATGCTGCTCTGGGAAACATGGAAGAGGACAACTGGAGATGGCATTTCTACGACACCGTGAAAGGATCTGACTGGCTTGGGGATCAGGATGCCATCCATTACATGACAGAGCAAGCCCCTGCCTCCGTGGTCGAGCTAGAAAATTATGGCATGCCATTTAGCAGAACTGAAGATGGGAAGATTTATCAGCGTGCATTTGGTGGACAGAGCCTCAAGTTTGGGAAAGGCGGGCAGGCTCATCGGTGTTGCTGTGTGGCTGATCGAACAGGCCACTCACTCTTGCACACCTTGTATGGAAGGTCTCTGAGATTTGACACCAGCTATTTTGTGGAGTATTTTGCCTTGGATCTTTTGATGGAAAATGGGGAATGCCGTGGTGTCATTGCACTGTGCATCGAAGATGGGTCTATACACCGAATAAGAGCAAAGAACACTGTTATTGCTACTGGGGGCTATGGGCGGACCTACTTCAGCTGCACATCTGCCCATACCAGCACAGGGGATGGCACAGCTATGGTCACCAGGGCTGGCTTACCTTGCCAGGACTTAGAGTTTGTTCAGTTCCACCCCACAGGTATATATGGTGCTGGCTGTCTCATCACAGAAGGGTGCCGTGGAGAGGGAGGTATTCTCATCAACAGTCAAGGTGAAAGGTTCATGGAGAGATATGCCCCTGTTGCCAAGGACCTGGCATCCAGAGATGTTGTATCTCGATCCATGACTCTTGAGATCCGTGAAGGAAGAGGCTGTGGCCCCGAGAAAGATCACGTCTACCTGCAGTTGCACCATCTGCCCCCTGAGCAGCTGGCCACACGTCTGCCTGGGATTTCAGAGACAGCCATGATCTTCGCTGGCGTGGATGTCACTAAGGAGCCTATCCCTGTCCTCCCCACTGTGCATTACAACATGGGTGGGATTCCCACTAACTACAAAGGGCAGGTGCTGAAGCATGTGAATGGACAGGATCAGGTTGTGCCTGGCCTGTATGCCTGTGGGGAGGCTGCCTGTGCTTCAGTGCATGGTGCCAACCGGCTTGGAGCAAATTCCCTATTGGACCTCGTAGTCTTTGGCCGAGCCTGTGCCCTGAGCATTGCAGAATCTTGCAGGCCTGGAGATAAAGTTCCTCCAATTAAAGCAAATGCTGGAGAAGAGTCAGTTATGAATCTTGACAAGTTAAGATTCGCTGATGGAAGCATAAGAACATCAGAACTACGCCTCAGCATGCAGAAGTCAATGCAGAATCATGCTGCAGTGTTCCGTGTGGGAAGTGTATTACAAGAAGGCTGTGAAAAAATTAGCCAGCTATATGGAGAACTGAAGCATCTAAAGACATTTGACAGAGGAATGGTCTGGAACACAGACCTGGTGGAGACCCTGGAGCTGCAAAACTTGATGCTGTGCGCACTGCAGACCATATATGGTGCAGAGGCACGAAAGGAGTCACGGGGAGCTCACGCCAGGGAAGATTATAAAGTGCGGGTTGATGAGTATGATTACTCCAAGCCAATCCAGGGGCAGCAGAAGAGGCCATTTGAGGAACACTGGAGGAAACACACCCTCTCATATGTGGACAACAACACTGGGAAGGTTACTTTGGAGTACAGACCTGTCATCGACAAGACCTTGAATGAAACTGACTGTGCCACAGTACCCCCTGCTATCCGTTCCTACTGATAAAACCTAGCAGCTACAGGACCAGCTTATGTGGTTATTTATCATAGCATACATAGGTTTACCTGTCATATTTGTCTTGTTAAAACTCTACTCTCATGAACAATGAGTCACTTCATAGATTATTGACCAACAGCTTGCTAGTATTTGATGTGAGGGACAGCTGCACCAGACTTTGTTGTTCCCTCCCCCCCACTTTTTTTTTTTTTGTGCAGTGATAAAACTGGCATAATTCTTAAATAAAATACAAACAAGCAACCTTTGTTTCCAAATAAAATCATGTGTTTGATTTTATGCTTATCTATTTGTTCAGACTAGGAAATTTTCTTCCCTGTCTAGTCCCAGCAGTTTTATCAAGTTTCTTGTGCTATATACTTGACTTTAGGGAATGTTAAGATGTAATGGTAAATAAAATTGAGAAGTAGGGTGGGCCCTTAATAATTGGGAAGAAAGTTTTGAACATGGTAGGTATAGAGCCAGTGCCTTGTTTAGGTTACTGGTGGGAAGAGATATATGCATATGTTCATGTATATGTGAGGGGGTTATATGCTGGTTAGCTTTGTCAACTTGACGAAATCTAGCATCACCTAGGAGGAGAGTCTCAATAAAGGATTGCTTCAATTTGA

>Drosophila_melanogaster (NM_057862.5)

AGCGAGTCAGTGAACCGAACTGCAGTGCCGACGAGCGGCAGTTCACACACAACATTTGAAAGTTTGTAAACTTTTCCTTTTTCGATCACATAGACACATCGATACACGTAGTTATTTGTTTTTATTTTGTACCAACACCAAACACCCCATCTTACAAGCGCGTAAAACCTCGTTTTCGGTCCAATCTCTTGCAGAATCTTGCAGTAGAACCTCACAGCCCAGCCAAACATGTCCGGAATCATGCGTGTGCCATCGATTTTGGCCAAAAATGCAGTCGCCTCCATGCAACGTGCCGCAGCCGTTGGAGTGCAGCGCAGTTACCACATCACACACGGCCGCCAGCAGGCCTCGGCGGCGAATCCGGACAAGATATCGAAGCAGTACCCGGTGGTGGATCACGCCTACGATGCGATCGTCGTGGGAGCAGGAGGAGCCGGCTTGCGGGCGGCTTTTGGCCTGGTGGCTGAGGGATTTCGCACGGCGGTGATCACCAAGCTTTTCCCCACCCGCTCGCACACGATCGCCGCCCAGGGCGGTATCAATGCCGCTCTCGGCAACATGGAGGAGGACGACTGGAAGTGGCACATGTACGACACGGTCAAGGGCTCCGATTGGCTGGGCGACCAGGATGCCATCCACTACATGACCCGCGAGGCGCCCAAGGCTGTCATTGAGCTGGAGAACTACGGCATGCCCTTCTCGCGTACCCAGGACGGTAAGATCTACCAGCGCGCCTTCGGTGGACAGAGTCTGAAGTTCGGCAAGGGCGGACAGGCCCATCGCTGCTGTGCTGTGGCTGATCGTACTGGTCACTCGCTGCTGCACACGCTATACGGTCAATCGCTGAGCTACGACTGCAACTACTTTGTGGAGTACTTTGCCCTGGATCTGATCTTCGAGGACGGCGAGTGCCGTGGTGTGCTGGCCCTGAACCTGGAGGATGGCACACTGCACCGATTCCGCGCTAAGAACACGGTCATTGCCACCGGTGGATATGGACGAGCATTCTTCTCCTGCACCTCGGCGCACACATGTACCGGTGACGGTACTGCTATGGTTGCACGCCAGGGACTGCCCTCTCAGGATCTGGAGTTCGTGCAGTTCCATCCTACTGGCATCTACGGCGCCGGATGTCTCATCACCGAGGGCTGCCGCGGTGAGGGTGGTTACCTGATCAACGGTAATGGTGAGCGCTTCATGGAGCGCTATGCTCCTGTGGCCAAGGATCTGGCCTCTCGCGACGTCGTCTCGCGGTCGATGACCATCGAGATCATGGAGGGTCGTGGCGCTGGACCCGAGAAGGATCACGTGTACCTGCAACTGCACCACTTGCCGCCCAAGCAGCTCGCGGAGCGTCTGCCTGGCATCTCCGAGACCGCCATGATCTTCGCCGGTGTTGATGTGACCCGTGAGCCCATCCCCGTGTTGCCCACCGTGCATTACAACATGGGCGGTGTGCCGACTAACTATCGCGGCCAGGTAATCACCATTGACAAGGATGGCAAGGATGTGATTGTGCCGGGACTGTATGCCGCTGGTGAGGCTGCTTCCAGCTCGGTGCATGGTGCCAACCGTCTGGGTGCCAACTCTCTGCTGGATCTGGTGGTCTTCGGACGTGCATGCGCCAAGACCATCGCCGAGCTGAATAAGCCTGGTGCACCGGCTCCCACCCTCAAGGAAAACGCTGGCGAGGCCTCTGTTGCTAACCTCGATAAGCTGCGCCATGCCAACGGCCAGATCACCACCGCCGATCTGCGTCTGAAGATGCAGAAGACCATGCAGCATCATGCTGCTGTGTTCCGCGATGGCCCCATCCTGCAGGACGGTGTGAACAAGATGAAGGAGATCTACAAGCAGTTCAAGGACATCAAGGTTGTCGATAGGTCGCTTATCTGGAACTCCGATCTGGTGGAGACGCTGGAGCTGCAGAATCTGTTGGCCAATGCCCAGATGACTATTGTGAGCGCTGAGGCGCGCAAGGAATCGCGAGGTGCCCACGCCCGCGAGGACTTCAAGGTTCGCGAGGATGAATACGATTTCAGCAAGCCCCTGGATGGTCAGCAGAAGAAGCCCATGGATCAGCACTGGCGCAAGCACACGCTCTCGTGGGTGTGCAATGACAACGGAGACATTACGCTGGACTACAGAAACGTGATCGATACCACGCTGGACAACGAAGTCTCTACTGTTCCACCAGCGATTCGCTCCTATTAAATCAAGACTTGGAGTTGCAGAACACCCGCATATACACCCCATACCATCACTCGTACGGATGTCTACTTAAACGTCTTTGTGGGCGCCAGTAAATAAATTTTGTCAAATCGCCAAATGAGCCGCAATATGCTACCAATTGGTAACTGGGAACTCGGCAGCGGTTCCGGGCATTTTGTGATGAAACGTAAATCCAGATTTTCTATACACCTATAAATCTTTCAAATCCTTATTACTACTCCGCTGATTCTACTTAGTTTCACTGTAAGCTGCCCTCCATTTGGTTACTGTTTGGTTGTCGGTTATTTACATTATTACATAAGCGAAAAATGTGTGTGAGTAGACAACGAGTTAGGTCTGAGGCGATATTGACTACGCAGCCTGTTTAGAAGCAAATGAAAACAAATCCAACTACACTTTTCTAGTATTTGTTAAACTTATTAAATTATTAAAACGAAAACGTGTAAAAACAATAAAAAAAGAACAAAAGTCGAAACGTTTGCTATACATTAAATAAATGTAATGAAGATTAAGTGCGC

>Homo_sapiens (NM_004168.4)

AGGGACTGGCGGGACTGCGCGGCGGCAACAGCAGACATGTCGGGGGTCCGGGGCCTGTCGCGGCTGCTGAGCGCTCGGCGCCTGGCGCTGGCCAAGGCGTGGCCAACAGTGTTGCAAACAGGAACCCGAGGTTTTCACTTCACTGTTGATGGGAACAAGAGGGCATCTGCTAAAGTTTCAGATTCCATTTCTGCTCAGTATCCAGTAGTGGATCATGAATTTGATGCAGTGGTGGTAGGCGCTGGAGGGGCAGGCTTGCGAGCTGCATTTGGCCTTTCTGAGGCAGGGTTTAATACAGCATGTGTTACCAAGCTGTTTCCTACCAGGTCACACACTGTTGCAGCACAGGGAGGAATCAATGCTGCTCTGGGGAACATGGAGGAGGACAACTGGAGGTGGCATTTCTACGACACCGTGAAGGGCTCCGACTGGCTGGGGGACCAGGATGCCATCCACTACATGACGGAGCAGGCCCCCGCCGCCGTGGTCGAGCTAGAAAATTATGGCATGCCGTTTAGCAGAACTGAAGATGGGAAGATTTATCAGCGTGCATTTGGTGGACAGAGCCTCAAGTTTGGAAAGGGCGGGCAGGCCCATCGGTGCTGCTGTGTGGCTGATCGGACTGGCCACTCGCTATTGCACACCTTATATGGAAGGTCTCTGCGATATGATACCAGCTATTTTGTGGAGTATTTTGCCTTGGATCTCCTGATGGAGAATGGGGAGTGCCGTGGTGTCATCGCACTGTGCATAGAGGACGGGTCCATCCATCGCATAAGAGCAAAGAACACTGTTGTTGCCACAGGAGGCTACGGGCGCACCTACTTCAGCTGCACGTCTGCCCACACCAGCACTGGCGACGGCACGGCCATGATCACCAGGGCAGGCCTTCCTTGCCAGGACCTAGAGTTTGTTCAGTTCCACCCTACAGGCATATATGGTGCTGGTTGTCTCATTACGGAAGGATGTCGTGGAGAGGGAGGCATTCTCATTAACAGTCAAGGCGAAAGGTTTATGGAGCGATACGCCCCTGTCGCGAAGGACCTGGCGTCTAGAGATGTGGTGTCTCGGTCCATGACTCTGGAGATCCGAGAAGGAAGAGGCTGTGGCCCTGAGAAAGATCACGTCTACCTGCAGCTGCACCACCTACCTCCAGAGCAGCTGGCCACGCGCCTGCCTGGCATTTCAGAGACAGCCATGATCTTCGCTGGCGTGGACGTCACGAAGGAGCCGATCCCTGTCCTCCCCACCGTGCATTATAACATGGGCGGCATTCCCACCAACTACAAGGGGCAGGTCCTGAGGCACGTGAATGGCCAGGATCAGATTGTGCCCGGCCTGTACGCCTGTGGGGAGGCCGCCTGTGCCTCGGTACATGGTGCCAACCGCCTCGGGGCAAACTCGCTCTTGGACCTGGTTGTCTTTGGTCGGGCATGTGCCCTGAGCATCGAAGAGTCATGCAGGCCTGGAGATAAAGTCCCTCCAATTAAACCAAACGCTGGGGAAGAATCTGTCATGAATCTTGACAAATTGAGATTTGCTGATGGAAGCATAAGAACATCGGAACTGCGACTCAGCATGCAGAAGTCAATGCAAAATCATGCTGCCGTGTTCCGTGTGGGAAGCGTGTTGCAAGAAGGTTGTGGGAAAATCAGCAAGCTCTATGGAGACCTAAAGCACCTGAAGACGTTCGACCGGGGAATGGTCTGGAACACGGACCTGGTGGAGACCCTGGAGCTGCAGAACCTGATGCTGTGTGCGCTGCAGACCATCTACGGAGCAGAGGCACGGAAGGAGTCACGGGGCGCGCATGCCAGGGAAGACTACAAGGTGCGGATTGATGAGTACGATTACTCCAAGCCCATCCAGGGGCAACAGAAGAAGCCCTTTGAGGAGCACTGGAGGAAGCACACCCTGTCCTATGTGGACGTTGGCACTGGGAAGGTCACTCTGGAATATAGACCCGTGATCGACAAAACTTTGAACGAGGCTGACTGTGCCACCGTCCCGCCAGCCATTCGCTCCTACTGATGAGACAAGATGTGGTGATGACAGAATCAGCTTTTGTAATTATGTATAATAGCTCATGCATGTGTCCATGTCATAACTGTCTTCATACGCTTCTGCACTCTGGGGAAGAAGGAGTACATTGAAGGGAGATTGGCACCTAGTGGCTGGGAGCTTGCCAGGAACCCAGTGGCCAGGGAGCGTGGCACTTACCTTTGTCCCTTGCTTCATTCTTGTGAGATGATAAAACTGGGCACAGCTCTTAAATAAAATATAAATGAACAAACTTTCTTTTATTTCCAAATCCATTTGAAATATTTTACTGTTGTGACTTTAGTCATATTTGTTGACCTAAAAATCAAATGTAATCTTTGTATTGTGTTACATCAAAATCCAGATATTTTGTATAGTTTCTTTTTTCTTTTTCTTTTCTTTTTTTTTTTTGAGACAGGATCGGTGCAGTAGTACAATCACAGCTCACTGCAGCCTCAAACTCCTGGGCAGCTCAGGTGATCTTCCTGACTCAGCCTTCTGAGTAGTTGGGGCTACAGGTGTGCACCACCATGCCCAGCTCATTTATTTTGTAATTGTAGGGACAGGGTCTCACTGTGTTGCCTAGGCTGGTCTCAAGTGATCCTCCCTCCTTGGCCTCCCAAGGTGCTGGAATTATAGGTGTGAACAAACCA

>Lonchura_striata_domestica (XM_021528737.1)

TCTGCCGGAGTCCCCCTTCCCCGCCGAGCCTGCGGTGCGCGATGGCGGCGGCGGCGGCTGCGCGGGGGCTGGCGAGGCGCTGGCTGCGCCCGGCCGCGGCTTCCCGGGCGTGGCCAGCAGCATGCCAGGCTCCTGCACGGAACTTACACTTCACAGCCTATGGAAAGAAAAATGCCTCCACGAAGGTGTCTGACTCGATTTCTACACAATACCCAGTAGTGGACCATGAGTTTGATGCAGTTGTTGTGGGTGCAGGAGGAGCAGGTCTGCGGGCTGCCTTTGGTTTGTCAGAAGCTGGGTTTAACACAGCCTGTGTTACCAAGCTGTTCCCCACCCGCTCTCATACTGTTGCTGCACAGGGAGGGATCAATGCAGCTCTGGGGAACATGGAGGATGATAACTGGAGGTGGCATTTCTATGACACAGTGAAAGGGTCGGACTGGCTGGGTGACCAGGATGCCATACATTACATGACTGAGCAAGCCCCAGCTGCGGTGATAGAGCTGGAAAATTATGGGATGCCGTTCAGCAGAACTGAAGAAGGAAAAATCTATCAGCGTGCGTTTGGTGGACAGAGTCTTCAGTTTGGAAAAGGAGGACAGGCCCACAGATGCTGTTGTGTTGCAGACAGGACTGGACATTCACTCTTACATACTCTGTATGGCAGGTCTCTACGATATGATACAAGCTACTTTGTTGAATATTTTGCCTTGGACTTACTTATGGAGAATGGAGAATGCCGTGGTGTTATTGCCCTCTGCATTGAAGACGGAACCATACATCGTTTTAGAGCAAAGAACACGGTCATTGCCACTGGTGGGTATGGCCGCACTTACTTCAGCTGCACGTCTGCTCATACCAGTACTGGTGATGGCACTGCCATGGTCACACGAGCTGGGCTCCCTTGCCAGGACTTAGAATTTGTGCAGTTTCACCCTACAGGTATCTATGGGGCTGGCTGCCTTATCACAGAAGGATGTCGTGGAGAGGGAGGTATTCTGATCAACAGTCAAGGTGAAAGGTTCATGGAGAGATATGCACCTGTTGCCAAGGATCTGGCTTCAAGGGATGTCGTGTCTCGTTCTATGACCATAGAAATCCGTGAAGGAAGGGGTTGTGGTCCTGAAAAAGACCATGTGTACTTGCAGTTGCACCATTTGCCACCAGAGCAGCTGGCAACCCGTCTCCCAGGGATTTCTGAAACAGCCATGATATTCGCTGGAGTTGATGTCACTAAAGAGCCCATCCCTGTCCTGCCTACTGTGCATTATAATATGGGAGGTATTCCTACTAACTACAAAGGCCAGGTGATCACACACGTGAATGGTGAGGATAAGGTGGTGCCCGGGTTGTACGCCTGCGGGGAAGCAGCCTCTGCATCCGTCCATGGCGCAAATCGGCTTGGAGCAAACTCCCTTCTGGATTTGGTGGTCTTCGGTCGTGCTTGTGCCCTCACTATTGCAAATACATGCAAGCCTGGAGAGCCAGTTCCCCCCATTAAACCAAATGCTGGTGAAGAGTCAGTTGCTAATCTTGACAAGTTACGGTTTGCTAATGGAAGCATAAGAACTTCAGAATTGCGACTTAACATGCAGAAGGCAATGCAAAACCATGCTGCTGTATTTCGCACTGGTTCTGTACTCCAAGAAGGCTGTGAGAAACTTAGCCAGATTTATGGTGATCTGGCTCATCTAAAGACTTTTGACAGAGGTATTGTGTGGAACACTGACTTGGTGGAGACACTTGAACTGCAGAACCTGATGCTTTGTGCTCTACAAACTATATACGGTGCTGAGGCTCGCAAAGAGTCACGGGGTGCCCATGCCAGAGAGGATTATAAGTTACGGGTGGATGAGTTTGACTACTCTAAGCCACTCCAAGGCCAGCAGCGGAAGCCATTTGAGGAGCACTGGAGAAAGCACACCCTGTCATATGTGGATATCCCAACTGGGAAGGTTACCTTGAAGTACAGACCAGTGATTGACAAGACATTGAATGAGGAAGATTGCCAGACCGTCCCACCAGCGATCCGCTCATACTAGCAGCTTCCATCTCACTTGAAGCCCTGTCCCAGAGGGGCCAGTGTACATAAGCATAGCACAAGCGAACCAGAATTCACTTAGCAATGAATAGAAAATGCTGACAAAATCTTCCTCCAGCTTGTATCCTGCTGTGGCTTACTGTACTTTTATTAAAAGTGGAATGCAGAACAGCTTATGTTCTTTCACTTATCTGTCAAAATGGACGGTGAAAACACAAAATGTGTCTAAATAAAGTAACTACAAGTCTTTTATTTGTTTGAA

>Oryzias_melastigma (XM_024268071.2)

TATATACCCCCATATTTACGGAACGGTCCAGTCCGGTCCCACAGGATATTTCTTCCCCACCAGCGGTTCAACATTCTGAGCTGGCAACATGGCGAGTGTTCGCGCTGCGTCTCGTCTCCTCTCCAGGAGGACGATTTCACACGTTAAAGCAGTTCCTGCTGCTGCCGTTCAAAGTTCAAGGAACTTTCACTTCTCCATCTACGGAAAAAAGAAGAATGCCAAAGTTTCAGATGACATATCCACTCAGTATCCAGTGGTGGACCACGAGTTTGACGCGGTGGTAGTGGGAGCAGGAGGAGCGGGGCTGCGGGCAGCTTTCGGCCTGTCCGAGGCCGGCTTTAACACCGCCTGCGTCACCAAGCTCTTCCCCACCAGATCTCACACTGTTGCTGCACAGGGTGGGATCAATGCGGCTCTGGGAAACATGGAGGACGACGACTGGAGGTGGCATTTCTATGACACAGTAAAGGGATCTGACTGGCTCGGAGACCAGGATGCTATCCATTACATGACGGAGCAGGCTCCCGCTGCTGTCGTGGAGTTGGAAAATTTTGGCATGCCGTTCAGCCGGACTGAAGACGGGAAGATCTACCAGAGAGCCTTCGGAGGTCAGAGTCTCAAGTATGGAAAAGGAGGTCAGGCTCATCGCTGCTGCTGTGTAGCAGATAGAACAGGACACTCGCTCCTGCATACTCTTTATGGAAGGTCCCTTCGTTATGACACCAGCTACTTTGTGGAGTACTTTGCTCTAGACCTGCTGATGGAAGATGGGCAGTGCAAAGGAGTGATAGCTCTCTGCATGGAGGATGGATCCATCCACCGCTTCAGAGCCCAAAACACCGTCATCGCTACAGGAGGTTATGGAAGAACGTATTTCAGCTGCACATCTGCCCACACAAGCACAGGAGACGGAAACGCCATGGTGACCAGAGCTGGCCTGCCCTGTCAGGACTTGGAGTTTGTGCAGTTTCACCCCACTGGGATCTACGGAGCCGGTTGCCTGATCACAGAAGGCTGCCGTGGTGAAGGAGGAATCTTGATCAACAGTGAGGGAGAGCGCTTCATGGAGCGCTACGCTCCCAACGCCAAAGACCTGGCCTCCAGAGACGTGGTGTCCCGCTCCATGACCATAGAGATCCGGGAGGGCAGGGGTGTCGGTCCAGAGAAGGACCACGTGTACCTGCAGCTTCACCACCTGCCTCCTCAGCAGCTGGCCACAAGGCTGCCCGGGATCTCGGAGACGGCGATGATCTTTGCTGGAGTGGACGTCACCAAAGAGCCCATCCCCGTCCTGCCCACAGTTCACTACAACATGGGAGGAATCCCCACAAACTACAAAGGACAGGTGATCGATTACACAGACGGTAAGGACACAGTGGTGCCTGGACTTTACGCCTGCGGTGAGGCAGCTTGTGCGAGCGTTCATGGCGCCAACAGGCTGGGCGCCAACTCCCTGCTGGACTTAGTTGTGTTTGGAAGAGCCTGTGCCCTCACTATTGCTAACGAGCACAAACCTGGAGAGAAGCTGTCCCCTCTGAAACCCAGCGCAGGAGAGGAGTCTGTGGCCAACCTGGACAAGCTCCGGTTTGCAAATGGAAGTCTGAGAACCTCTGAGATCCGACTTAACATGCAGAAGACCATGCAGAACCACGCTGCAGTTTTCCGTACCGGCTCCGTCCTCAAAGAAGGATGCGATAAGATGGACGACATTTACCAGACCATGGAGCAAATAAAGACCTTTGACAGAGGCATTGTGTGGAACACAGACCTGGTGGAATCCTTGGAGCTGCAGAACTTGATGCTGAATGCGGTTCAGACCATCAACTCTGCCGAGCAGAGAAAGGAGAGCAGAGGAGCCCACGCTAGAGAGGACTTCAGAGATCGCGTGGATGAGTTTGATTACTCCAAGCCGCTGCAGGGTCAGGAGAAGAAACCCTTCGACCAGCACTGGAGGAAGCACACGCTGTCATACGTCGACCCCAAAACTGGAAAGGTGACCCTGAAGTACCGTCCCGTCATCGACACCTCCCTGGATGAGCAGGACTGCGCTCACGTTCCCCCCGCCATCCGATCCTATTAAAATCTCAAACCCATCCACGCTCACTTACTCCTTACTCAGTTACAGAATGTTAATCATACTGCTCTTCAATAAAATCCTGTCTGTAGCAAGAGATTACGATGTAGAACAGTCAGTGGCGTTCATTTGCAGATGTTAAACGCAGTGTCGTCTCATCAGCTTCCGGTGCTTCGTGTCACTCGTGTCTCACCATCCAGTTTGGCTTTTAGTGGCATCGCATGCGATGTACTAAATGTTTGAGTAATTCTGACGTTAACTCCAGAGCGAAGAGGCCATGAGAGTGATTGTTGTTCGATTTGTCCGTCGCTGAGACAGAAGGAAGACTTTAAAGTATGTAAATATTGCACAAGCAATGTGGTGACCCTCACCGCCTCTCACTCCCTGCAGCTTTTTAGGTTTTAAAAGTATTTACTGATCAAACATAATATTCTGCGCATGGCTCAGTTTCAGCTGGTCATCATTAAGCCTGCTCATGTGTTTTGTTGTTGATGTGAGGAGATTTTGAAATAAAAAGGGCAGTAAATGCTACAGTGCACAGGAATGAATTTGGATTTAAGAAAAAATAAACTGTTCTGAGAGTTCCCTGTGTTTGTGTGGCATTGCATCAGAAATCTTAGCATTTATATCCACGGTTGTTGTTATGAATCAATAACGAGGGTGTTTTAATTTATTTGAAGAACAGTTAAAAAATATAATTGTTTTGCCATTTGTACTGGTTTCCATACATTCAATAAAGTGTATGCTATGCTCACAAACAAAAA

>Gekko_japonicus (XM_015420735.1)

GCCGACCGGCTGCCGGCAGGGGCGAAATGGCGGCGGCGGCCGTGAGGGGATGGCGGCGACCTTTGCGGGCGTTCCGCTGGCCGGGGCCAGCGGCCTGCCAGGCCTTCTCTCGTCAGTTCCACTTCACAGTTTACGGAAAGAAAAGTGCATCTGCTAAAGTATCGGATTCAATTTCAACTCAGTATCCCGTCGTGGACCACGAATTTGATGCCGTGGTGGTGGGCGCAGGAGGTGCCGGGCTGCGAGCTGCATTTGGCTTGTCTGAGGCTGGCTTTAACACAGCATGTGTTACCAAGCTCTTCCCTACCAGGTCCCATACTGTCGCTGCACAGGGAGGAATCAATGCTGCTCTGGGGAACATGGAAGAAGATAACTGGCGGTGGCATTTCTACGACACGGTGAAAGGCTCGGACTGGCTGGGGGACCAGGATGCCATTCATTACATGACGGAGCAGGCCCCGGCTGCCGTTATTGAGCTGGAGAAATACCTGAAGTTTGGGAAAGGGGGGCAGGCCCATCGCTGCTGCTGCGTGGCTGACCGGACTGGCCACTCCCTGCTGCACACGCTCTACGGCCGGTCTCTTAGATATGATACTAGCTACTTTGTGGAATATTTTGCCTTGGACCTCCTTATGGAAAACGGAGCATGTCACGGCGTTGTGGCCCTTTGCATAGAAGATGGCACCATCCATCGTTTGAGAGCAAAGAATACGGTCCTTGCCACTGGAGGATACGGGCGCACCTACTTCAGCTGCACATCTGCTCACACCACCACTGGAGACGGCACCGCAATGGTCACACGGGCCGGCCTTCCCTGCCAGGACCTGGAGTTTGTGCAGTTCCATCCTACAGGAATCTATGGGGCTGGCTGCCTTATTACGGAAGGCTGTCGTGGTGAAGGAGGCATTCTCATTAACAGTGAAGGCGAAAGGTTCATGGAGAGATATGCGCCAGTCGCCAAGGACCTAGCTTCAAGGGATGTTGTATCTCGTTCTATGACCATCGAGATCCGTGAAGGAAGAGGATGTGGGCCCGAGAAGGACCACGTGTACCTGCAGCTGCATCACCTGCCTCCGCAGCAGCTAGCGATGCGTCTCCCGGGGATCTCGGAGACGGCCATGATATTTGCTGGGGTGGATGTGACGAGGGAGCCCATTCCTGTCCTGCCCACGGTGCATTACAATATGGGGGGGATCCCGACTAACTACAAAGGACAGGTGATCACTCATGTCAATGGCCAGGATCAGGTCGTGCCAGGCTTGTATGCTTGTGGCGAAGCGGCATCTGCGTCCGTCCACGGGGCCAACCGCCTGGGGGCAAACTCTCTCTTGGACTTGGTGGTCTTTGGCCGCGCCTGTGCTCTTAGTATTGCGGAATCCTGCAAGCCCGGAGAACCAGTTCCGTCAGTTAAACCAAATGCAGGGGAAGAATCTGTCGCAAACCTGGACAAATTGCGCTTTGCCAATGGAAACCTAAGGACATCAGAACTGAGGCTTAATATGCAGAAGACCATGCAAACACATGCCGCTGTGTTCCGTACGGGCTCTGTCCTCCAGGAGGGTTGTGAGAAACTGAGCAGCATCTACAAGGATCTGGACAATCTGAAGACATTTGACAGAGGGGTGGTCTGGAATACTGACTTGGTGGAGACTTTGGAACTTCAAAACCTGATGCTTTGTGCGTTGCAAACTATTTATGGTGCCGAAGCTCGGAAAGAGTCGCGTGGTGCTCACGCAAGGGAGGACTACAAGGTTAGGATAGATGAATATGACTATTCCAAACCAACTGAAGGACAACAGAAGAAACCATTTGATCAGCACTGGAGAAAGCACACCCTCTCGTATGTCGATGTCGGGACTGGGAAGGTTACTTTGGAGTACAGACCTGTCATCGACAACACTCTAAATGAAGAAGACTGTGCCATGGTCCCACCTGCTATTCGTTCCTATTAGTGATTTAATGCACCTTTTTGTTATCTGGAGGCTGCGTGTTATGGCTGATCATCTTACTAGACAACCGTTACGCACAATAGTGGATGAAATTGTCATCTTTGAGAGTCTGTTCCATAGCACAGAAGAGAGGCCCATTGCTTCACGGGTGATAAAACCTAGCAATATTCAACTCATGCCTCAATAAAGTAATCTACCAGTATGTACACACCTGAGTCAATTTCTGGTTTTCTTTGAATATCAGGCTCTGCACACAAGGGTTTTATCTCTTGCTTCTGACTATGCTTTTTGTATTGATTCTCTTAAACATTTGAAAGGGTAAATGCAATGTGATTATTGAGAATTGGCTTCTGTTGAAATGAAACAACATTTTAGGGCACAGTTCAGTGGACCTAAATGTGCTTAAGCACCAAACCACTGGCTTGTTGCCTTAATTTTATATCTCTGCCTTCTACCTTGTATGCTGTTTAATTTGTGGCAATTTTGGCATGAACTGTGAAGTTTACAAAAGTACCATAGTACACATGTTAAAGGTTAGTAGGACTTCCCTGTAACACACTTAAGTATTTCTTCTCTTTCAAGGTAATACAGTATATCGTTGGACATGTGGGAGTTTATACCATTATAATATTCTTAACGGACATCTTAAACTAATTTTCTTTTCTGTTACTGTTCATTTGACCCCGTGGTTCCGAACAGCCTCTGAGAGTTTGTTTAAGCAACTAATGGAAAGCTACCCACAAAATGGGATCTCCCCAACAAAATTTGATAGGGTTTATGCAGTTGTTTTAGAATGGTAAACACTAATATTCATGGTAAGAGAAGGTTGTTGGGAATATATCCCTAGAAAGGGCCGGACTGTATGAAACAGGTTTTCTTTCTGTCAGATGTATAATACCAATGGAGCTTCAGTTCAGAACATGCACTGTATGTTAAAAAAACAAAAACTCTCTCCTAAATATACGCTGCTCTTATTTAATGCATATTATTGTAAGTTATATTATATTTTGTATTGTACAAGTTCAGCCGATTAAGCAGCAAAAGAATTGAAGAACGGTTTTGTAGCCTCCGAGACAATTATACGAATCTCTCCGTTAACTTTCCTGAATGTGTCTCCTTTCTCCAGCGGCTCAGCCATCTTCCTGTGATGAGAACTGTGGCACATGCAGAGCAAAATCTCTTCCGTTGTCTTTGCTGTGCACGAGTAAGAATGCTCTGGGTTGGTTTCAGACCTCTTCAGTGGGAACCCTGGTGGTGGTGGTGGGAAGTGCCTGCCGTCAAACTGCTACTGATGTA

>Neophocaena_asiaeorientalis_asiaeorientalis (XM_024731953.1)

GCGGCGACGTGCGCAGGCGCGGTCTGCGCGGGGACTGGCGGGACGGTTGCGGAGGCAGACATGTCGGGGATCGGGGTTTTGTCGCGGATGCTTCGCGCCCGGCGCCTAGCGCTGACCTGGGCGCAGCGGCCAGAAGCATTGCACGCAGGAGCCCGCAGTTTTCACTTCACCGTTGATAGCAGTAAGAGGTCGTCCGCTAAAGTTTCAGATGCAATTTCTACTCAGTACCCCGTAGTGGATCATGAATTCGATGCAGTGGTGGTCGGCGCCGGAGGGGCAGGCCTGCGAGCTGCATTTGGCCTTTCTGAAGCAGGGTTTAACACGGCCTGCGTCACAAAGCTCTTTCCCACCAGGTCACACACCGTCGCAGCCCAGGGAGGGATCAACGCCGCCCTGGGGAACATGGAGGAGGACAACTGGAGGTGGCACTTCTACGACACCGTGAAGGGCTCCGACTGGCTGGGGGACCAGGATGCCATCCACTACATGACGGAGCAGGCCCCGGCCTCCGTGGTGGAGCTGGAGAATTACGGCATGCCGTTTAGCAGAACTGAAGATGGGAAGATCTACCAGCGTGCCTTCGGTGGACAGAGCCTCAAGTTTGGGAAAGGCGGGCAGGCCCATCGGTGCTGCTGCGTGGCCGACCGCACTGGCCACTCGCTGCTGCACACTTTGTATGGAAGGTCTCTGCGATATGATACCAGCTATTTTGTGGAGTATTTTGCCTTGGACCTCCTGATGGAGAACGGGGAGTGTCGAGGCGTTATAGCTCTGTGCATAGAAGACGGGTCCATCCACCGCATCAGAGCAAAGAACACTGTTGTAGCTACTGGAGGCTACGGGCGCACCTACTTCAGCTGCACATCCGCCCACACCAGCACCGGGGACGGCACTGCCATGATCACCAGGGCAGGCCTTCCCTGCCAGGACCTGGAGTTTGTTCAGTTCCACCCCACAGGCATATACGGTGCTGGCTGTCTCATCACGGAAGGGTGCCGTGGAGAGGGAGGCATCCTTGTCAACAGTCAAGGCGAGAGGTTCATGGAGCGCTACGCTCCTGTCGCAAAGGACCTGGCATCCAGGGACGTCGTATCCCGCTCCATGACCCTGGAGATCCGTGAAGGAAGAGGCTGTGGCCCTGAGAAGGATCACGTGTACCTGCAGCTGCATCACCTCCCCCCGGAACAGCTGGCTATGCGCCTGCCTGGCATCTCGGAGACAGCCATGATCTTCGCGGGTGTGGACGTCACCAAGGAGCCGATCCCCGTGCTTCCCACCGTGCACTATAACATGGGCGGCATCCCCACCAACTACAAGGGGCAGGTTCTGAAGCACGTGGGCGGCCAGGACCAGGTCGTGCCCGGCCTGTACGCGTGTGGGGAGGCTGCCTGCGCGTCAGTGCACGGCGCCAACCGGCTGGGGGCGAACTCGCTCCTGGACCTGGTCGTCTTCGGCCGGGCGTGTGCGCTGGGCATCGCGGAGTCCTGCAAGCCCGGAGATAAAGTTCCCTCGATTAAACCAAATGCTGGGGAAGAATCTGTCATGAATCTTGACAAATTGAGATTTGCCAACGGAAGCATAAGAACGTCGGAACTACGGCTGAACATGCAGAAGTCGATGCAAAGTCATGCCGCAGTGTTCCGTGTGGGAAGCGTGTTACAGGAAGGCTGTGAGAAGATCAGCAAGCTCTATGGAGATCTAAAGCATCTGAAGACGTTTGACAGGGGAATGGTCTGGAACACTGACCTGGTGGAGACCCTGGAGCTGCAGAACCTGATGCTTTGTGCCCTGCAGACCATTTATGGAGCAGAGGCCCGGAAGGAGTCTCGCGGCGCTCACGCCAGGGAGGATTTCAAGGAGCGGGTTGACGAGTACGATTACTCCAAGCCCATCCAGGGGCAGCACAAGAGGCCGTTTGAGGGGCACTGGAGGAAGCACACGCTCTCCTACGTGGACATCAGGACTGGGAAGGTCTCCCTGGAATACAGACCCGTAATCGACAGAACTTTGAACGAGGCTGACTGCGCCACTGTCCCCCCAGCCATCCGCTCCTACTGAGGAGACCGGAGTTTACCAGCTCTTGTAATTATGTATAATAGCTCATGCACATGTTCATATTGTAACTGCCTTCTTAAGCTGTCCTCGAGTGACTGAGGATGCCGCTTGACAGAGATTCCTACCAGAGGCCGGCAGCTCGCCAGTAATCAACAGCCAGGAAGTGTTCAGCTTGCCTTTGTCCCTGCTTCATTCTTGAGAGATAATAAAACTGGGCGCAATTCTTAGATGAAACATAAGTTACAAACTTGCGTTGATTTCCAAGTTCCTTTAAAATCTTACTGTTTGACCTGAGGCATATTTGTTGACCTGAAACATAAAACGTGACTATCTTTTTATTCTTTTGCATCAAAATCCAGAAATTTTGCTGCAGTGTATGTTTAAGTGAAAATGCATTTTGGTGCCTGACAGCAAATGAAGAAAACAGACGTTTCCTCCTAGTTCTTAGGAAGCAAATGGTTTAACATGTGAACGTGTGTCCTGAAGTATTTAAATGACCGAATACTAAATAAAACTGATGTGGGTGCAAATGGG

**OCEANIA**

>Crocodylus_porosus (NC_008143.1)

ATGTTAAAAATCATTGTACCCACAATAATACTAATTCCCTCAACCTGCCTGACAGCCACAAAAAACACCTGATTATCGCCAACAGCCTACTCAGCAGTTATTATTATCCTAGGCATACTTGTCTTAAACCCCGGAGACACCCTGATGAACACTACTGGTCTACTACTAGGAAGTGACCAAATCTCAACACCCTTACTTATACTATCCTGCTGACTACTACCACTAATATTTATGGCTAGCCAAAGCTCCATGTCACACAACCCTGCCCAACAAAAACGACTGTTTATCACAGCCCTAGCCCTCCTACAATTAGCCTTAATATTAGTATTCATGGCCTTAGACCTAATGTTATTCTACACCACCTTTGAAGCAACCCTTATTCCCACCCTAATAGTGATCGCCCGATGGGGATCCCAAACAGAACGACTCGGAGCCGGACTATATTTCCTCCTATACACCATCACTAGCTCCATACCCCTTCTAATCGCACTTCTATGGGTGTATAACATAAAAGGAACTGCGTCTATTACACTCTTACAGCTACTCCCCCCAATAACCCTAACATTCTGAACAAACACGCTACTATGAACCTCACTCATATTGGCCTTCCTAGTAAAAATCCCAATTTACGGCCTCCACCTTTGGCTACCAAAAGCCCACGTAGAAGCCCCAATTGCCGGATCCATGGTCCTTGCAGCAATCTTATTAAAACTCGGGGGTTATGGCCTGCTACGAATTACAAACCTATTAACTGAACAAACTACATCCTCTTATATTCTCCCACTGGCAGTAGCACTATGGGGTGCACTCATAACCGGCATAGTCTGCCTACGACAAACAGATTTAAAATCCCTAATTGCCTACTCCTCAGTAAGCCACATAGGACTAATAACAAGCTCGATCCTCACTCACAATCAACTAGCCCCATCAGGATCAATAATTATAATAGTGGCCCACGGCCTTACATCTTCGATACTATTCTGCCTGGCAAATATTAATTACGAACGAACACACTCACGAACCCTATTACTTACACAAGGGGTACAACTAACCACCCCAGCCATGACGTCCTGATGACTCCTAGCCTGCTTAACAAACATAGCACTTCCCCCAACAATTAATTTCATTGGGGAACTCACCCTTATAGTCTCACTATTTGACTGAGCAGACATTACTATCTTTCTAACAGGACTAAGCGCATTCATCACCTCAATCTACACCCTACACATATTCTCCTCAACCCAACAGGGAACCCTTCCAACCCATATTATTACAATAAGTCCAACCCAAACTCGAGAACATCTACTAATAACACTGCACTCCGCACCATCAATCGCTTTAATCTTTATACCTCAACTAATATACTACCAATAA

>Drosophila_melanogaster (NC_024511.2)

ATGTTAAAAATTATTTTTTTTTTATTATTTTTAATTCCTTTTTGTTTTATTAATAATATATATTGAATGGTACAAATTATAATATTTTTTATTAGATTTATTTTTTTATTAATAAATAATTTTATAAATTATTGATCAGAAATTTCTTATTTTTTAGGTTGTGATATATTATCTTATGGATTAATTTTATTAAGTTTATGAATTTGTTCATTAATATTATTAGCTAGAGAAATAATTAATAAACATAATAATTATAAAAATTTATTTTTATTAAATATTATTATTTTATTATTATTGTTGATTTTAACTTTTTCAAGAATAAGATTATTTATATTTTATTTATTTTTTGAAAGAAGACTAATTCCTACATTATTTTTAATTTTAGGTTGAGGTTATCAGCCAGAACGTTTACAAGCTGGTTTATATTTATTATTTTATACTTTATTAGTTTCTTTACCTATATTAATTGGTATTTTTTATTTAATAAATAAAATTGGTTCTATAAATTTTTATTTAATAAATAATTTTATGTTTAATTACGATTTATTATATTTTTGTTTATTGTGTGCCTTTTTAGTAAAAATACCAATATTTTTAGTTCATTTATGATTACCTAAAGCTCATGTTGAAGCTCCAGTTTCTGGGTCTATAATTTTAGCAGGTATTATATTAAAATTAGGAGGTTATGGAATATTACGAGTTATTAGTTTTTTACAGTTAATAAATTTGAAGTATAGATTTGTTTGAATTAGAATTAGATTAGTAGGAGGTGTATTAGTTAGATTAGTTTGTTTACGTCAAACTGATTTAAAGGCTTTAATTGCTTATTCATCTGTTGCTCATATAGGAATTGTTCTATCAGGACTTTTAACTATAACTTATTGAGGTTTATGTGGTTCTTATACATTAATAATTGCTCATGGTTTATGTTCTTCTGGGTTATTTTGTTTAGCTAATGTATCTTATGAACGTCTTGGAAGTCGAAGAATATTAATTAATAAAGGTTTATTAAATTTTATACCTTCGATAACTTTATGATGATTTTTATTAAGATCAGCTAATATAGCAGCTCCTCCAACATTAAATTTATTAGGAGAAATTTATTTATTAAATAGAATTGTTTCTTGATCTTGAATTTCTATAATTTTATTATCTTTTTTATCTTTTTTTAGAGCTGCTTATACTTTATATTTATATTCTTTTAGTCAGCATGGTAAATTATTTTCTGGAGTATATTCATTTAGAAGAGGTAAAATTCGAGAATATTTATTAATATTGTTACATTGATTGCCTTTAAATTTATTAATTTTAAAAAGAGAATCATTTATATTATGATTAT

>Homo_sapiens (NC_012920.1)

ATGCTAAAACTAATCGTCCCAACAATTATATTACTACCACTGACATGACTTTCCAAAAAACACATAATTTGAATCAACACAACCACCCACAGCCTAATTATTAGCATCATCCCTCTACTATTTTTTAACCAAATCAACAACAACCTATTTAGCTGTTCCCCAACCTTTTCCTCCGACCCCCTAACAACCCCCCTCCTAATACTAACTACCTGACTCCTACCCCTCACAATCATGGCAAGCCAACGCCACTTATCCAGTGAACCACTATCACGAAAAAAACTCTACCTCTCTATACTAATCTCCCTACAAATCTCCTTAATTATAACATTCACAGCCACAGAACTAATCATATTTTATATCTTCTTCGAAACCACACTTATCCCCACCTTGGCTATCATCACCCGATGAGGCAACCAGCCAGAACGCCTGAACGCAGGCACATACTTCCTATTCTACACCCTAGTAGGCTCCCTTCCCCTACTCATCGCACTAATTTACACTCACAACACCCTAGGCTCACTAAACATTCTACTACTCACTCTCACTGCCCAAGAACTATCAAACTCCTGAGCCAACAACTTAATATGACTAGCTTACACAATAGCTTTTATAGTAAAGATACCTCTTTACGGACTCCACTTATGACTCCCTAAAGCCCATGTCGAAGCCCCCATCGCTGGGTCAATAGTACTTGCCGCAGTACTCTTAAAACTAGGCGGCTATGGTATAATACGCCTCACACTCATTCTCAACCCCCTGACAAAACACATAGCCTACCCCTTCCTTGTACTATCCCTATGAGGCATAATTATAACAAGCTCCATCTGCCTACGACAAACAGACCTAAAATCGCTCATTGCATACTCTTCAATCAGCCACATAGCCCTCGTAGTAACAGCCATTCTCATCCAAACCCCCTGAAGCTTCACCGGCGCAGTCATTCTCATAATCGCCCACGGGCTTACATCCTCATTACTATTCTGCCTAGCAAACTCAAACTACGAACGCACTCACAGTCGCATCATAATCCTCTCTCAAGGACTTCAAACTCTACTCCCACTAATAGCTTTTTGATGACTTCTAGCAAGCCTCGCTAACCTCGCCTTACCCCCCACTATTAACCTACTGGGAGAACTCTCTGTGCTAGTAACCACGTTCTCCTGATCAAATATCACTCTCCTACTTACAGGACTCAACATACTAGTCACAGCCCTATACTCCCTCTACATATTTACCACAACACAATGGGGCTCACTCACCCACCACATTAACAACATAAAACCCTCATTCACACGAGAAAACACCCTCATGTTCATACACCTATCCCCCATTCTCCTCCTATCCCTCAACCCCGACATCATTACCGGGTTTTCCTCTT

>Lechriodus_melanopyga (NC_019999.1)

ATGCTAACTGCAGTTTTTGCCTTTTTAACTATAATCCTAACTACATATCTAGCCCCGACCAACCTATTATGGGCGCTAGTTACGGCTCAATCCTTTATTATTGCTGTCCTGTCATTTCTATGATTCACATCATCAGAAATCCTCCCAACACTTAACCAATACCTGCTTGTTGACGAAATCTCTTCCCCTCTTCTCATCTTAACCTTCTGACTAACCCCCCTAACACTTTTAGCAAGCCAAAGCAAAATCTCTAAAGAGCCGGCCTCCCGACAACGATCATATATCTTTTCCATCCTATTTCTTCAAATTACAACTGCCCTGGCCTTCTTAGCTAACAACCTCATACTATTTTTCATCTTTTTTGAGTCCACTATAATTCCAACCTTAATTGTTATTACCCGCTGAGGAACTCAAAAAGAACGAATCATAGCAGGTATTTACATGCTCTTTTACACCCTCGCCGGGTCTCTTCTCCTCTTAACAGCCATTCTTTATTTCCACGAATCTTTTGGTTCACTTACTATTTCTATACTAAAAAAAATTCAAGAAGAACAAATAATCAACTTCTCCTCTACAATCTGGTGACTAGCCTGCCTTGTAGCATTCCTAGTTAAAATACCCCTGTACGGGCTCCACCTCTGGCTCCCTAAAGCCCACGTCGAAGCTCCAATCGCCGGCTCAATAATTTTAGCCGGTACCCTTTTAAAACTGGGGGGCTACGGCCTTCTTCGTATATCTACACTAATTAATGAGTCTATATACGCCCTTGCCACCCCTTTAATTATTTTCTCCATATGCGGAGTCGTGGCCTCAGCACTCCTCTGTTCACGCCAAACCGATCTAAAATCTTTAATTGCCTTTTCGTCAGTTAGTCACATGGGCCTTGTTGTAGCCGCATCCATGATTAAATCACCATGAAGCGTCTCTGGAGCAATAATTCTAATAATCTCCCACGGCCTTATTTCCTCCGCCCTGTTTTGCCTAGCCAACACAGCATATGAACGAACCAGCACTCGAACTATAGTCGTCCTTCAAGGGAGCCAAGTTATACTCCCCCTCGCTGCAGCATGATGGCTCCTGTCAGTTATACTAAATATGGCCCTCCCCCCTTCCACCAACTTCATGGGAGAACTCCTAATTTTACAATCAATTTTCAAATGGGCTCCGTACTCTATTATTCTAGCTGGCCTTGGTATTATTTTCACCACATCTTACTCCCTCTACCTTTTTTGATCCTCACAACGAGAGCATCTCCCCACTCACTTAAAGTCGATACCCCCGACTGTAACTCGGGAACACATTCTTTTAGCACTGCACATCTTACCCATCATTTTTTTAATCATCAAGCCTGAGCTAATCTTTTAA

>Maccullochella_macquariensis (NC_028167.1)

ATGCTAAAAATCCTTATCCCAACCTTAATACTAATCCCAACAGCCTACACAACCAAAGCCAAATGACTATGACCCACAACACTCTTCTACAGCCTAACTATCGCCCTCACAAGCCTTCCTCTATTAAAAAACCTCTCAGAAACAGGCTGATCTTCACTTGGATTATATATAGCAACAGACAATCTATCAACCCCTCTCCTAATCCTCACCTGCTGACTTCTTCCACTTATAATCCTTGCAAGCCAAAAACACACAACCTTCGAACCCATTAACCGCCAACGAACATACATCGCACTACTAACATCTCTACAACTCTTCCTAATCCTAGCCTTTAGCGCAACTGAACTTATCATATTTTATATCATATTTGAAGCCACCTTGATTCCAACCTTAATCCTAATCACACGCTGAGGAAACCAAATAGAACGACTCAACGCCGGCACCTATTTTCTATTTTATACACTAGCAGGCTCTTTACCATTACTAGTTGCTCTTATACTCATCCAAAAAAATACAGGAACACTATCTCTATTAACCCTTCAATACTCAAACCCAGTCCCAATACTAACATACGCAGATAAACTATGATGAGCAGGTTGCTTACTAGCCTTTCTAGTGAAAATACCACTATACGGCGTACACTTATGACTACCTAAAGCACACGTTGAAGCCCCTATTGCAGGCTCTATAATTCTGGCTGCAGTACTTTTAAAACTAGGAGGTTACGGTATAATACGAATAATAGTAATATTAGAACCACTAACAAAAGAATTAAGCTACCCATTCCTTATTTTCGCACTCTGAGGCGTTATTATAACAGGATCCATTTGCCTCCGCCAAACAGACTTAAAATCCCTAATCGCATACTCATCAGTTAGTCATATAGGCCTAGTTGCAGGCGGTATTTTAGTACAAACTTCTTGAGGATTCACAGGAGCCCTTATTCTTATAATCGCCCATGGACTAACATCTTCCGCCCTATTTTGCCTAGCAAACACCAATTATGAACGTACCCATAGTCGAACCTTAATACTAACCCGTGGACTACAAATAGCACTTCCACTCATAACCACATGATGATTCATCACTAGCCTTGCCAACCTAGCACTTCCCCCTCTCCCTAATCTAATTGGAGAACTAATAATCATCATTTCCTTATTTAACTGGTCCTGATGAACCATTGCTTTAACAGGAACCGGAACCCTTATCACAGCAGGCTACTCCCTATATATATTCCTCACAACCCAACGAGGCCAACTCCCCACACACATTCTCACCATAGACCCCTCCCACACACGAGAACACCTACTAATCACCCTCCACCTTCTTCCACTAATCCTGCTCACCTTCAAACCAGAATTAATCTCAGGTTGAACCTCCT

>Phascolarctos_cinereus (NC_008133.1)

ATGCTAAAAATCCTTATACCAACATTCATATTAATCCCACTTACCTGACTATCCAAAAACCTATGACTATGAACCAACCTCACATCATACAGCCTATTAATTGGAACCTTCAGCATTACACTTCTACATCAAGACTCAGACCTAGGCACAAACCATAATAACCTATTTTATACAGACTCATTATCTAGCCCCTTACTAGTTCTATCATGCTGACTACTTCCCCTAATAATAATAGCAAGTCAAAATCACCTAAACAAAGAAAGCATAAACCGAAAAAAAGCTTACCTGACCACACTAATTATTCTACAACTATCACTAATTACAGCTTTATCAGCATCAGAACTCATAATATTCTATATCATATTCGAAACAACCCTTATCCCCACCCTAATTATTATTACACGATGAGGAAACCAAAATGAACGACTAAACGCTGGACTTTACCTTCTATTTTATACCCTAACAGGATCAATTCCCCTCTTAATTGCCCTACTATTCTTATATAACAAACTAGGATCATTACATATTCTAGCCATAACTATTATATCAACCACCCTAAAACCTTCTTACTCTAATTCAATCCTATGATATGCATGTATAACCGCATTCATAGTTAAAATACCTCTATACGGCCTACACCTATGACTGCCAAAAGCACACGTAGAAGCACCAATCGCAGGATCAATAGTACTAGCAGCTATTCTACTAAAATTAGGAGGCTATGGAATTATACGAATAACAGTCTTTACCCAGCCCCTAACCACAAACCTTTACTACCCATTCATTATCCTATCACTATGAGGAATAGTCATAACAAGCTTCATCTGCTTACGCCAAACAGACCTAAAATCTCTAATCGCCTACTCCTCAATCAGCCACATAGCTCTAGTAATCATCGCTACTCTTATACAGTCACCACTAAGCTTCATAGGTGCTACAGCCCTAATAATTGCCCACGGACTGACATCCTCTATATTATTCGGCCTAGCCAACACAAACTACGAACGTATCAACAGCCGAACTATAATCTTAGCTCGAGGACTACAAACTATTCTCCCACTAACATGCACCTGATGAATCTTAGCCACTTTAGCCAATCTAGCTCTCCCACCAACTATCAACTTCTTATGCGAATTACTAGTAATCACCTCATCCTTCTCTTGATCAAACTTTTCCATCATCTTACTGGGTATTAACACTATTATTACAGCACTTTACTCACTTCACATACTAACAACTACACAACGAGGAAAACCTACATACCATACACAAACTATTAAACCAACCTCCACACGAGAACACACCCTAATAATCCTCCACCTAATTCCACTACTAACCATCTCATTAAGTCCTAAATTCATCCTAGGACTCACATACT

>Sus_scrofa (NC_000845.1)

ATGCTAAAAATTATTATCCCAACAACAATACTACTACCCATAACATGAATATCTAAACACAACATAATCTGAATCAATGCAACAGTACATAGTCTCCTCATTAGCCTGATCAGTCTATCCCTACTAAACCAACTAGGCGAAAACAGCCTTAATTTTTCCTTAACATTCTTCTCCGACTCACTATCAGCACCCCTACTAGTTCTAACCACATGACTCCTCCCCCTTATACTAATAGCTAGCCAATCTCACCTATCAAAAGAAACCACAACCCGAAAAAAACTATATATTACCATACTAATCCTACTACAACTATTCCTAATTATAACCTTCACCGCCACCGAACTAATCCTATTCTATATCCTATTCGAAGCAACACTAGTACCCACACTAATTATCATCACACGCTGAGGAAACCAAACAGAACGACTCAATGCAGGACTTTATTTCCTATTCTACACCCTAGCAGGATCCCTACCACTGCTAGTAGCACTAGTTTATATCCAAAATACCACAGGCTCACTAAACTTCTTAATTATCCATTACTGATCCCACCCATTATCCAACTCTTGATCAAACATTTTTATATGATTAGCATGCATCATAGCCTTCATAGTAAAAATACCTCTGTACGGACTCCATCTTTGACTGCCAAAAGCCCATGTAGAAGCCCCCATTGCAGGTTCAATAGTACTTGCAGCCGTACTGCTAAAACTCGGAGGCTATGGCATAATGCGAATCACTACTATTCTAAACCCACTAACAAACTACATAGCCTATCCATTCCTCATGCTTTCCATATGAGGCATAATCATAACCAGCTCTATCTGCTTACGTCAAACCGACCTAAAATCCTTAATCGCCTATTCATCAGTAAGTCATATAGCACTTGTAATCGTAGCAATCATAATTCAAACCCCTTGAAGCTTCATAGGGGCCACAGCTCTCATAATTGCCCACGGACTAACATCCTCCATACTATTCTGCCTAGCCAACACTAACTATGAACGAGTACACAGCCGAACCATAATCCTGGCCCGAGGACTGCAAACACTCCTACCACTCATAGCAACATGATGACTAATAGCAAGCCTCACAAACCTAGCCCTACCCCCATCCATCAATCTAATCGGAGAATTATTTATCATCACAGCATCATTTTCATGATCCAACATCACAATTATTCTCATAGGAATAAACATAATAATTACAGCCCTCTACTCTCTCTACATACTAATTACTACACAACGAGGAAAATACACCCACCACATTAACAACATCAAAGCCTCATTCACACGAGAAAACGCCCTCATAGCCCTACATATTCTACCACTACTACTACTGACCTTAAACCCTAAAATAATCCTAGGACCCCTTTACT

>Tregellasia_capito (NC_027231.1)

ATGCTAAAAATCCTCATCCCAACTGCCATACTCCTACCCCTAGCACTCTGCTCCCCGCGCAAACATTTATGAACCAACACAACAGCATACAGCCTACTAATCGCCGCAGCCAGCTTACAATGACTCACTCCCACCTACTACCCAAACAAAAACCTGTCCAACTGAGCTGCTATCGACCAAATCTCCTCCCCCCTACTAGTTCTCTCATGCTGACTACTCCCACTAATAATTATAGCAAGCCAAAATCACCTAGAACAAGAACCCACCATCCGTAAACGAATTTTCATCACAACATTACTTCTAGCTCAACCCTTCATTCTTACTGCCTTCTCAGCTTCAGAGCTAATACTATTCTACATCGCATTCGAAGCCACCCTAATCCCCACCCTAATCTTAATTACACGATGAGGGAGCCAACCAGAACGACTAACCGCTGGCATCTACCTTCTATTCTACACTCTCGCCAGCTCCCTACCCTTGCTCATCGCTATCCTACACCTACAAAACCAAATCGGCTCACTATCCTTTATAATACTTAAACTAACACATCCAACAATAACCTCCTCCTGAACAAGCTTAGTAACCGGCCTGGCCCTACTCCTGGCCTTCATAGTAAAAGCACCCTTATACGGCCTACACCTATGACTGCCCAAAGCCCATGTAGAAGCCCCAATCGCCGGCTCCATGCTTCTTGCAGCCCTACTTTTAAAACTAGGAGGGTACGGAATCATACGAGTTACCATACTGGTAAACCCCTCATTAAACAACCTACACTATCCATTTATCACCCTAGCCCTATGAGGAGCACTAATGACTAGCACCATTTGCCTACGACAAATCGACCTAAAATCACTAATCGCCTACTCATCTGTAAGCCACATAGGACTAGTCGTAGCTGCAACCATAATCCAAACCCAATGAGCCTTTTCAGGCGCAATAATATTAATAATCTCACATGGCCTGACCTCCTCTATACTATTCTGCTTAGCTAATACCAACTACGAACGCACACACAGCCGAATCCTCCTACTAACACGAGGACTACAACCCCTCCTCCCCCTTATAGCCACCTGATGACTTCTAGCCAACCTAACAAACATAGCCCTCCCCCCAACAACCAACCTCATAGCAGAACTTACCATCGCAATCGCCCTATTCAACTGATCCTCCCTTACAATCATTCTAACAGGAGGTACAATCCTACTAACCACCTCATACACCCTATACATACTCACAATGACACAACGAGGAACAATCCCACCCCACATCACATCTATCCAAAACTCCTCTACACGAGAACACCTCCTCATGGCCTTACACGTAATCCCCATAGCCCTACTCATCCTTAAACCCGAGCTAATCGCAGGCATCCCCATATAG

**AFRICA**

>Acinonyx_jubatus (NC_005212.1)

ATGACCAACATTCGAAAATCACACCCCCTTATCAAAATCGTTAATCACTCATTCATCGATTTACCCACCCCACCTAACATTTCAGCATGATGAAACTTCGGCTCCCTACTAGGAGTCTGCCTAGTCCTACAGATCCTAACCGGCCTTTTCCTAGCCATACACTACACATCAGACACAATAACCGCCTTTTCATCAGTTACTCACATCTGCCGCGACGTCAACTACGGCTGAATTATTCGATACATGCACGCCAACGGAGCCTCTATATTCTTTATCTGCCTATACATGCATGTAGGACGAGGAATATACTACGGCTCCTACACCTTCTCAGAAACATGAAATATTGGAATCATACTACTACTCACAGTCATAGCCACAGCCTTCATAGGATATGTCTTACCATGAGGTCAAATATCTTTCTGAGGAGCAACTGTAATTACCAACCTCCTATCAGCAATTCCTTACATCGGCACTAATCTAGTAGAGTGAATTTGAGGAGGTTTCTCGGTAGACAAAGCTACCCTGACACGATTCTTTGCCTTCCACTTTATCCTTCCATTCATCATCTCAGCCCTAGCAGCAGTACACCTCTTATTTCTCCACGAGACAGGATCCAATAACCCCTCAGGAATCACATCCGACTCAGACAAAATTCCATTCCACCCATACTACATAATCAAGGACATTCTAGGTCTCCTAATACTAATTTTAATACTCACACTACTCGTCCTCTTCTCACCAGACCTGTTAGGAGATCCAGACAACTACATCCCCGCCAACCCCCTAAACACTCCTCCCCATATTAAACCCGAATGATATTTTTTATTCGCATACGCAATCCTCCGATCCATCCCTAACAAATTAGGAGGAGTCCTAGCCCTAATATTCTCCATCCTAATCCTAGCAATCATTCCAATTTTCCACACCTCCAAACAACGAGGAATAATATTTCGACCACTAAGCCAATGCTTATTCTGACTCCTAGTAGCAGACCTCCTCACCCTAACATGAATTGGCGGCCAACCTGTAGAACACCCCTTTATTACCATCGGCCAACTAGCCTCCATTCTATACTTCTCAACCCTCCTAGTTCTAATACCTATTTCAGGCATCATCGAAAACCGCCTCCTAAAATGAAGA

>Drosophila_melanogaster (NC_024511.2)

ATGAATAAACCTTTACGAAATTCCCATCCTCTATTTAAAATTGCCAATAATGCTTTAGTAGATTTACCAGCTCCAATTAATATTTCAAGATGATGAAATTTTGGATCATTACTTGGATTATGTTTAATTATTCAAATTTTAACCGGATTATTTTTAGCTATACATTACACAGCTGATATTAATCTAGCTTTCTATAGTGTTAATCATATTTGTCGAGACGTTAATTATGGTTGATTATTACGAACTTTACATGCTAACGGTGCATCATTTTTTTTTATTTGTATTTACTTACATGTAGGACGAGGAATTTATTACGGTTCATATAAATTTACTCCAACTTGATTAATTGGAGTAATTATTTTATTTTTAGTAATAGGAACAGCTTTTATAGGATACGTATTACCTTGAGGACAAATATCATTTTGAGGAGCTACTGTAATTACTAATTTATTATCAGCTATCCCTTACTTAGGTATAGATTTAGTTCAATGATTATGAGGTGGATTTGCTGTTGATAATGCCACTTTAACTCGATTTTTTACATTCCATTTTATTTTACCTTTTATTGTTCTTGCTATAACTATAATTCATTTATTATTCCTTCATCAAACAGGATCTAATAATCCTATCGGATTAAATTCTAATATTGATAAAATTCCTTTTCATCCTTATTTTACATTTAAAGATATTGTAGGATTTATTGTAATAATTTTTATTTTAATTTCATTAGTATTAATTAGACCAAATTTATTGGGAGACCCTGATAATTTTATTCCAGCAAATCCTTTAGTAACACCTGCCCATATTCAACCAGAATGATATTTTTTATTTGCTTATGCTATTTTACGATCTATTCCAAATAAATTAGGAGGAGTTATTGCATTAGTTTTATCAATTGCAATTTTAATAATCCTTCCTTTTTATAATTTAAGAAAATTCCGAGGGATTCAATTTTATCCTATTAATCAAGTAATATTCTGATCTATATTAGTAACAGTAATTTTATTAACTTGAATTGGAGCTCGACCAGTTGAAGAACCTTATGTATTAATTGGACAAATTCTAACTGTTGTATATTTCTTATATTATTTAGTAAACCCATTAATTACAAAATGATGAGATAATTTATTAAATTAA

>Homo_sapiens (NC_012920.1)

ATGACCCCAATACGCAAAACTAACCCCCTAATAAAATTAATTAACCACTCATTCATCGACCTCCCCACCCCATCCAACATCTCCGCATGATGAAACTTCGGCTCACTCCTTGGCGCCTGCCTGATCCTCCAAATCACCACAGGACTATTCCTAGCCATGCACTACTCACCAGACGCCTCAACCGCCTTTTCATCAATCGCCCACATCACTCGAGACGTAAATTATGGCTGAATCATCCGCTACCTTCACGCCAATGGCGCCTCAATATTCTTTATCTGCCTCTTCCTACACATCGGGCGAGGCCTATATTACGGATCATTTCTCTACTCAGAAACCTGAAACATCGGCATTATCCTCCTGCTTGCAACTATAGCAACAGCCTTCATAGGCTATGTCCTCCCGTGAGGCCAAATATCATTCTGAGGGGCCACAGTAATTACAAACTTACTATCCGCCATCCCATACATTGGGACAGACCTAGTTCAATGAATCTGAGGAGGCTACTCAGTAGACAGTCCCACCCTCACACGATTCTTTACCTTTCACTTCATCTTGCCCTTCATTATTGCAGCCCTAGCAACACTCCACCTCCTATTCTTGCACGAAACGGGATCAAACAACCCCCTAGGAATCACCTCCCATTCCGATAAAATCACCTTCCACCCTTACTACACAATCAAAGACGCCCTCGGCTTACTTCTCTTCCTTCTCTCCTTAATGACATTAACACTATTCTCACCAGACCTCCTAGGCGACCCAGACAATTATACCCTAGCCAACCCCTTAAACACCCCTCCCCACATCAAGCCCGAATGATATTTCCTATTCGCCTACACAATTCTCCGATCCGTCCCTAACAAACTAGGAGGCGTCCTTGCCCTATTACTATCCATCCTCATCCTAGCAATAATCCCCATCCTCCATATATCCAAACAACAAAGCATAATATTTCGCCCACTAAGCCAATCACTTTATTGACTCCTAGCCGCAGACCTCCTCATTCTAACCTGAATCGGAGGACAACCAGTAAGCTACCCTTTTACCATCATTGGACAAGTAGCATCCGTACTATACTTCACAACAATCCTAATCCTAATACCAACTATCTCCCTAATTGAAAACAAAATACTCAAATGGGCCT

>Erpetoichthys_calabaricus (NC_005251.1)

ATGGCCATCATACGAAAAACCCACCCTTTAGCCAAAATCATTAATAGCGCATTTATTGACCTACCAGCACCATCAAATATCTCTTCATGATGAAATATGGGATCTCTTCTAGGACTATGTCTAATTGTACAAATTATTACAGGATTATTCCTGGCAATACATTATATTTCAGATATTAACTTGGCCTTTTCATCCGTGGCTCACATTTGCCGAGACGTTAATTACGGCTGACTTATTCGCAACATTCATGCTAACAGCGCATCTCTATTTTTTATCTGTATTTACCTACACATTGCACGCGGCCTATATTATGGTTCGTATTTATATATAGAAACATGAAATGTAGGAGTTATCCTATTACTTCTGACTATAATAACCGCATTCGTGGGGTATGTATTACCTTGAGGCCAAATATCATTCTGAGGTGCAACTGTCATTACAAACTTATTATCAGCAGTTCCTTATATCGGAGATACCTTAGTTCAGTGAATTTGAGGGGGGTTTTCCGTAGACAAACCAACATTAACTCGATTTTTTGCTTTCCATTTTATCCTGCCATTTGCAATTGCAGGAGCATCCTTAGTCCACATCTTATTCCTCCACGAAACGGGCTCTAATAACCCACTAGGAATTAATTCAAACGCAGATAAAATCCCGTTCCACCCATATTATACATATAAAGACCTTCTAGGATTTATTATCTTATTACTAATTATTTTAATATTAGCCCTACTCTCCCCCAACTTACTAAACGACCCAGAAAATTTTACACCGGCCAACCCCCTAGTAACCCCACCTCATATTAAACCAGAATGATATTTTCTATTTGCCTACGCTATTCTACGCTCAATTCCAAATAAACTAGGAGGAGTATTAGCCCTGCTATTCTCTATTATTGTATTAATATTTGTACCATTTTTACACACTGCAAAAATTCGAACTTCCACATTTCGCCCTCTATTTAAAATTACATTATGAATTCTAGCCGCTGATGTTATAATCTTAACATGAATTGGAGGACAGCCAGTAGAAGATCCTTACATTATAATTGGACAAGTGGCTTCAGTACTTTACTTTACTATCTTCCTAGTATTTATACCAGTATCCGGTTGAATTGAAAACAAAATAATGAACCGCAACT

>Fukomys_damarensis (NC_027742.1)

ATGACCAACATCCGAAAATCCCACCCCTTAATCAAAATTATCAACCACTCGTTCATCGACCTGCCCACACCCTCCAGCATCTCCTACTGATGGAACTTCGGATCACTCCTAGGAGCCTGCCTAATCCTACAAATTATCACAGGTCTATTCCTATCCATGCACTACACTGCAGACACAGCCACCGCATTCTCATCAGTAGCCCACATCTGCCGAGATGTAAACTACGGATGATTAATCCGATACTTACACGCCAATGGTGCCTCCATATTTTTCATCTGCCTGTATCTTCACGTAGGTCGAGGAATATACTACGGATCTTACATATTCATAGAAACCTGAAACATCGGAATCATTCTTCTCCTATCAGTTATAGCAACTGCTTTCATAGGATATGTGCTACCATGAGGACAAATATCATTCTGAGGTGCGACAGTCATCACAAACCTATTCTCAGCAATCCCTTACATCGGCCCAACACTAGTAGAATGAATCTGAGGCGGATTCGCAGTAGACAAAGCCACTCTGACCCGATTCTTCGCCTTCCACTTCATCCTACCGTTCATCATCACAGCACTTACGATAGTACACCTGCTATTCCTACACGAAACTGGATCAAACAACCCATCAGGCATCAACTCAGACTCGGACAAAATCCCATTCCACCCCTACTACTCATTTAAAGACTTCATAGGATTGCAGATCATACTACTCATTCTACTAACACTAACACTATTCCACCCAGACCTACTAGGAGACCCCGATAACTACACACCAGCAAACCCCATGAGCACGCCACCACACATCAAACCAGAATGATATTTCCTGTTTGCATACGCCATCCTACGCTCTATTCCCAACAAACTAGGAGGAGTACTAGCCTTGGTTATATCAATCCTGATCCTAGTCGCCCTACCACTCCTACACACATCCAAACAACGCAGCATAATATTCCGACCTATCAGCCAATGTTTATTCTGAACGTTCATCTCCACCCTCCTCACCCTAACATGAATCGGCAGCCAACCAGTAGAATACCCTTACATCATCATCGGCCAATTAGCATCAATCCTATACTTCCTCATCATCCTTGTTCTAATACCCTTAGCAGGGTTAGTAGAAAATAAAATAATAAAATGAAGA

>Numida_meleagris (NC_034374.1)

ATGGCCCCCAACATTCGAAAATCTCACCCCCTATTGAAAATAATTAACAACTCCCTAATCGACCTTCCTACCCCATCAAACATCTCTGCCTGATGGAACTTCGGATCCCTCCTAGCAGTCTGCCTCATGACCCAAATTATCACCGGCCTACTACTAGCTATACACTACACTGCAGATACCTCCCTAGCCTTCTCATCCGTAGCCCACACATGTCGAAATGTCCAATACGGATGACTAATCCGAAACCTACATGCAAACGGAGCCTCATTCTTCTTCATCTGCATCTACCTCCACATTGGCCGAGGCCTATACTACGGCTCCTACCTATATAAAGAAACCTGAAACACAGGAGTAATTCTCCTCCTCACACTAATAGCAACCGCTTTCGTAGGCTACGTTCTTCCATGAGGCCAAATATCATTCTGAGGGGCTACTGTCATTACTAATCTATTCTCAGCTATCCCCTACATTGGACAAACTCTAGTAGAGTGGGCGTGGGGAGGGTTTTCAGTCGACAACCCCACCCTCACTCGATTTTTCGCCCTACACTTCCTTCTCCCCTTCGTCATCGCAGGAATCACAATTATCCACCTCACATTCCTTCACGAATCGGGCTCAAACAACCCCCTAGGCATTTCATCCAACTCAGACAAAATCCCATTCCACCCCTACTACTCCATCAAAGACATCCTAGGCCTAACACTTATACTCACCCCACTCCTAACCCTAGCCCTATTCTCCCCAAACCTACTAGGTGACCCCGAAAACTTTACCCCAGCCAACCCACTAGTAACACCCCCACACATCAAACCAGAATGATACTTCCTATTTGCATACGCCATCCTCCGCTCAATTCCAAACAAACTTGGAGGCGTACTAGCACTAGCAGCCTCCGTACTTATCCTCCTCCTAATCCCATTCCTCCACAAATCCAAACAACGAACCATAACATTCCGCCCATTCTCCCAACTTCTATTCTGACTCCTAGTAGCCAACCTTCTCATTCTAACCTGAGTGGGCAGCCAACCTGTAGAACACCCCTTCATCATCATCGGACAACTAGCATCACTCTCCTACTTCACTACCCTCCTAATCCTCTTCCCCATAATTGGAACCCTAGAAAACAAAATACTTAACCACTAA

>Xenopus_laevis (NC_001573.1)

ATGGCACCCAACATCCGTAAATCTCATCCATTAATTAAAATTATTAATAATTCTTTCATTGACCTCCCAACCCCATCAAACATTTCATCATTATGAAACTTCGGCTCTCTTCTAGGGGTCTGTTTAATTGCCCAAATCATTACAGGATTATTCTTAGCTATACATTATACAGCAGACACATCTATAGCCTTCTCATCAGTAGCCCATATTTGTTTTGACGTTAACTATGGATTATTAATTCGCAATCTCCATGCCAATGGACTCTCATTCTTCTTCATTTGCATCTACCTTCACATCGGACGAGGGTTGTACTACGGCTCTTTCTTATATAAAGAAACATGAAATATTGGTGTGATCCTCCTATTTTTAGTTATAGCTACAGCATTTGTAGGATATGTTCTACCATGAGGACAAATATCTTTTTGAGGGGCTACAGTAATTACTAATCTTCTTTCTGCTAAACCGTACATCGGAAACGTACTAGTCCAATGAAGTTTAGGAGGATTCTCTGTAGATAACGCCACTTTAACCCGATTCTTCGCATTTCACTTCCTCCTTCCTTTTATTATTGCCGGAGCTAGCATTCTCCATCTTTTATTTCTCCACGAAACTGGATCAACAAACCCAACTGGATTAAACTCAGACCCAGATAAAGTACCTTTCCACCCATACTTCTCTTACAAAGACCTTTTAGGCTTCCTTATTATACTTACAGCACTTACTCTCCTAGCCATATTTTCCCCAAACCTTTTAGGAGACCCAGACAATTTTACCCCAGCTAATCCTCTAATCACCCCTCCACATATTAAACCAGAATGATACTTCCTATTCGCCTACGCTATCCTTCGATCCATAAACAAACTAGGCGGAGTGTTAGCCCTAGTCCTATCCATCCTAATCTTAGCCCTCATACCATTACTCCACACATCAAAACAACGAAGCCTTATATTCCGACCATTTACACAAATCATATTTTGAGCCCTAGTTGCAGATACACTAATCCTAACCTGAATTGGAGGTCAACCAGTAGAAGACCCTTATACCATAATTGGACAGTTAGCCTCAGTAATTTACTTCTCAATCTTTATTATTATATTCCCACTTATAGGTTGAGTAGAAAATAAACTATTAAACTGATAG

>Giraffa_giraffa (EF442270.1)

ATGATCAACATCCGAAAGTCCCACCCACTAATAAAAATCGTAAATAACGCACTAATCGATCTACCAGCCCCATCAAATATCTCATCATGATGAAACTTCGGCTCCCTACTAGGCATCTGTCTCATCTTACAAATCCTAACAGGCCTATTTCTAGCAATACACTACACACCTGACACAACAACAGCATTCTCCTCTGTCACCCATATTTGCCGAGATGTCAACTACGGTTGAATCATCCGATACATACACGCAAATGGGGCATCCATATTTTTCATCTGCCTATTCATGCACATGGGGCGAGGCTTGTACTATGGGTCATACACCTTTCTAGAAACATGAAACGTCGGGATAATCCTCCTATTTACAGTAATAGCCACAGCATTCATAGGGTACGTTCTACCATGAGGACAAATGTCATTCTGAGGCGCAACAGTCATCACCAATCTCCTATCAGCAATCCCATATATCGGCACAAACCTAGTCGAATGAATCTGAGGAGGCTTCTCAGTAGACAAAGCAACCCTTACCCGATTCTTCGCCTTCCACTTCATTCTCCCATTCATCATTGCAGCACTCACTATAGTCCACCTACTCTTCCTTCACGAAACAGGATCAAACAACCCAATAGGAATCACATCAGACATAGACAAGATCCCGTTCCACCCCTACTACACTATCAAAGACATCCTAGGAGCACTTCTATTGATTCTAACCCTAATACTTCTAGTCCTATTTACACCCGACTTGCTCGGAGACCCAGACAACTACACCCCAGCAAACCCACTCAACACACCCCCTCATATTAAACCCGAGTGATACTTTCTATTCGCATATGCAATCCTACGATCAATCCCCAATAAACTAGGAGGAGTCCTAGCCCTAATCTTCTCCATCCTCATTCTCGCTCTCATACCCCTACTCCACACATCCAAACAACGAAGTATGACATTTCGACCACTCAGTCAATGCTTATTTTGAATTCTAGTAGCGGATCTGTTAACACTCACATGAATTGGAGGACAGCCAGTTGAACACCCATTCATCATTATCGGACAACTAGCATCTATTATGTATTTTCTCATCATCCTAGTGTTAATACCAGTCACTAGTGCGATCGAAAACAACCTTCTAAAATGAAGA
