# Supplementary material for: Using active learning methodologies to teach sequence analysis and molecular phylogeny
Source: Biochem Mol Biol Educ. 2024 Oct 14;53(1):21–32. doi: 10.1002/bmb.21861 (PMC11752413; doi:10.1002/bmb.21861)
Supplement: Supplementary file 8 — Data S4. Doc.4 Supplementary material. The most common errors and learning difficulties encountered by the students in the preparation of the final report. [file BMB-53-21-s003.docx]

**A document for students which identifies and analyses the most common errors and learning difficulties in the preparation of the final report.**

This document identifies elements of the report that require further attention. The objective is to enhance the content and format of an upcoming report in order to optimise its effectiveness and ensure that it meets the required standards.

1. It is not permissible to simply copy headings from the results or objectives section in the abstract. It is about synthesising the entire content of the work, from the introduction to the conclusions.
2. The introduction and objectives sections have been competently executed. Please note that the text should be written in your own words and not copied verbatim. This will assist you in comprehending the concepts and in the practice of scientific writing. Additionally, it is advisable to exercise caution when translating into English, as the accuracy of Google Translate is not always guaranteed.
3. The Materials and Methods section displays a diversity of content. The objective was to provide a detailed account of the instruments employed, the initial materials utilized, and a step-by-step procedure, thereby enabling any individual to replicate the methodology. A common error has been to describe BLAST as a sequence translator. It should be noted that it is a sequence alignment program that compares a given problem sequence against a multitude of sequences stored in a database.
4. In the Results section, most of you have simply presented tables and figures without any accompanying explanation. It is essential to provide a detailed description of the images, including the relevant information beyond the mere identification of the images by name, which was occasionally absent. It is essential that the reader is aware of the nature of the material they are examining and its provenance. It is essential that figures and tables are numbered, titled and referenced consistently in the text in accordance with the prescribed format. Furthermore, they must be presented in a legible size font.
5. In the Discussion section, the results should be elucidated and integrated in order to draw conclusions from the study. A significant proportion of you have approached the task in a manner akin to that of a questionnaire, providing answers to the questions posed in the model. This was not the objective of the questions. As previously stated in class, the questions were designed to prompt ideas for discussing your own results, in a manner analogous to that employed in a scientific article. To facilitate this process, we provided models of articles in both English and Spanish.

Considering the discussions presented in the reports, it is important that you undertake a comprehensive study and review of the following:

- The unifying factor among the captured sequences may be attributed to either their geographical origin or their status as orthologous proteins. Some of you provided an incorrect and incoherent response, identifying *Drosophila melanogaster* as the unifying factor.
- It is important to understand the distinction between orthologous and paralogous genes. All genes are homologous, sharing a common evolutionary origin and exhibiting similar sequences. Orthologous genes are those that show homology across different species. Paralogous genes, on the other hand, are those that have arisen from a single gene through duplication within the same species.
- The degree of conservation of the protein or gene is contingent upon the number of differences between the sequences of the various species under study. A greater degree of conservation is indicated by a smaller number of differences, whereas a lesser degree of conservation is indicated by a larger number of differences. This can be deduced from the similarity and identity parameters provided by BLAST during alignment, the image obtained in the alignment performed with the SeaView program, the study of protein function, etc.
- A molecular clock candidate must meet several conditions to be considered suitable. Firstly, it must be distributed universally. Secondly, it should not be subject to horizontal transmission. Thirdly, it must possess functional constancy, so that the selective pressure acting on the molecule is minimal. Fourthly, the sequence length should be sufficient for statistical validity of similarity estimates. Fifthly, the rate of change of at least part of the molecule should be low enough to allow detection of distant evolutionary relationships. Sixthly, from a methodological point of view, it is of interest that it should not be excessively long to apply sequencing techniques.
- Regarding the interpretation of phylogenetic trees, there has been considerable confusion. As previously discussed in class, the ordering of branches in phylogenetic trees is intended to facilitate comparison and interpretation. The order of the branches does not determine the evolutionary relationships between species. It is necessary to comment on these relationships (how they are grouped) by determining the common ancestors, more or less recent, of the same, taking into account the specific analysis being conducted, in this case the sequences of a single gene or protein. The reference phylogeny created with morphological data is useful for comparing results from two different approaches. However, as many students have indicated, it is not a reliable method for determining whether the results are accurate or not.
- The results are a matter of fact; any errors made are likely to be procedural or in the initial approach. Once identified, it is important to attempt to explain the outcome based on the available evidence.
- This brings us to the concluding section, the bibliography. In order to gain this knowledge, it is necessary to engage in rigorous study and investigation of the material presented in class. To achieve this, it is essential to consult reliable sources of information, such as scientific articles and textbooks. It is notable that the majority of students have not included any bibliographic references beyond those drawn from the database and Wikipedia. This results in two fundamental shortcomings: 1) Inadequate discussion and erroneous conceptualisation; 2) Insufficient adherence to scientific writing conventions. It is not sufficient to write a scientific paper in the style of a casual conversation with a friend.
